# Exploring the Relationship Between Respiratory Syncytial Virus and Asthma Using Calendar Time at Birth as a Source of Quasi‐Random Variation

**DOI:** 10.1111/ppe.70168

**Published:** 2026-08-20

**Authors:** Michelle C. Dimitris, Daniel R. Feikin, Erin Sparrow, Susan E. Schultz, Jeffrey C. Kwong, Sarah A. Buchan, David A. Savitz, Steven M. Brunwasser, Deshayne B. Fell

**Affiliations:** ^1^ Children's Hospital of Eastern Ontario Ottawa Ontario Canada; ^2^ World Health Organization Geneva Switzerland; ^3^ ICES Ontario Toronto Ontario Canada; ^4^ Public Health Ontario Toronto Ontario Canada; ^5^ Dalla Lana School of Public Health, University of Toronto Toronto Ontario Canada; ^6^ School of Public Health, Brown University Providence Rhode Island USA; ^7^ Department of Psychology Rowan University Glassboro New Jersey USA; ^8^ School of Epidemiology and Public Health, University of Ottawa Ottawa Ontario Canada

**Keywords:** asthma, causal inference, natural experiment, quasi‐experiment, respiratory syncytial virus

## Abstract

**Background:**

Observational studies suggest an association between severe respiratory syncytial virus (RSV) and asthma, but it is unclear if this relationship is causal or confounded.

**Objectives:**

We investigated this relationship using calendar time at birth as a source of quasi‐random variation with respect to individual‐level risk factors for severe RSV infection. We similarly examined influenza (another respiratory virus) and rotavirus (a negative control exposure).

**Methods:**

We analysed province‐wide linked administrative data for 1,437,731 infants born in Ontario, Canada, from 2002–2013 (which facilitated 5 years of follow‐up for asthma). We ascertained whether each infant experienced an RSV, influenza, and/or rotavirus hospitalisation (and the weekly incidences of RSV, influenza, and rotavirus hospitalisation among infants), and whether each child had asthma at 5 years of age. We used regression models to investigate the relationships between: (i) infant ages during the weeks with the highest incidences of RSV, influenza, and rotavirus hospitalisation and paediatric asthma; (ii) weekly incidences of RSV, influenza, and rotavirus hospitalisation and probability of paediatric asthma.

**Results:**

We observed the highest odds of asthma among infants aged 13–16 weeks during the RSV peak, 11–13 weeks during the influenza peak, and 16–19 weeks during the rotavirus peak. The relationship between weekly incidence of RSV hospitalisation and probability of paediatric asthma was small in magnitude; we estimated a 3.15 (95% confidence interval −0.07, 6.36) percentage‐point change in probability of paediatric asthma per 1 percentage‐point change in weekly incidence of RSV hospitalisation among infants (offset by 13 weeks, which was the infant age at RSV peak that conferred maximum crude odds of paediatric asthma). Additionally, an unexpected relationship between rotavirus seasonal variation and asthma emerged.

**Conclusions:**

We found evidence supporting a limited causal role that did not fully explain the relationship between RSV and asthma. Other mechanisms, including seasonal factors, may contribute to previously reported associations.

## 1 Background

Respiratory syncytial virus (RSV) infects 50%–70% of infants and causes approximately 33 million cases per year of RSV lower respiratory tract infection (LRTI) among children aged ≤ 5 years [1, 2]. Although conditions like preterm birth and lung/heart disease increase susceptibility to severe RSV LRTI, most cases occur among relatively healthy infants [3, 4, 5, 6, 7, 8]. Observational studies describe an association between severe RSV LRTI and subsequent asthma [9, 10, 11], but are limited in their ability to infer causality given the potential for unmeasured confounding. Accordingly, two non‐mutually exclusive hypotheses have emerged: (i) severe RSV LRTI and paediatric asthma share common causes; and/or (ii) severe RSV LRTI causes paediatric asthma [11].

Evidence consistent with the first hypothesis includes the following: there is insufficient evidence that monoclonal antibodies (mAbs) for RSV prophylaxis are associated with a reduction in physician‐diagnosed or pulmonary function test‐defined asthma in a randomised controlled trial [12, 13] and observational studies [14, 15, 16]; among twins, there is insufficient evidence that discordant RSV hospitalisation in infancy/early childhood is associated with discordant asthma development [17, 18]; adjusting for genetic influences attenuates the association between RSV LRTI and asthma [16]; lower neonatal lung compliance and/or higher resistance is associated with both subsequent RSV hospitalisation and wheeze [19]; and some studies report insufficient evidence that RSV viral load and/or disease severity is associated with wheeze/asthma [20, 21].

Conversely, evidence that supports the second hypothesis includes the following: randomised receipt of mAbs is associated with reduced parent‐reported wheeze and asthma [12, 13]; mAbs for RSV prevention are associated with reduced wheeze in preschool‐aged children [22, 23, 24]; and some studies report that RSV viral load and/or disease severity is associated with wheeze/asthma [25, 26, 27, 28]. Recently, a prospective cohort study among term and non‐low birthweight infants found that RSV infection (measured by active and passive surveillance) in the first year of life was associated with non‐atopic asthma at 5 years of age. The study's focus on RSV infection (regardless of illness severity) is notable because it is less susceptible to confounding by genetic factors [29].

Studies that leverage ‘quasi‐random’ variation in the risk of RSV infection (and subsequent LRTI) can investigate causality. For example, investigators in the United States examined the association between infant age during their first winter respiratory virus seasonal peak (a hypothesised source of ‘quasi‐random’ variation in infant risk of RSV LRTI) and paediatric asthma [30]. The investigators concluded that infants born approximately 4 months before this peak were at the highest risk for asthma diagnosis at 5 years of age [30]. Moreover, studies using population‐based data in Sweden and Taiwan found a higher risk of childhood asthma (< 7 and 7–15 years of age, respectively) among those born in specific months (September–February and August–January, respectively) [31, 32]; the Sweden‐based study concluded that LRTI partially mediated this relationship.

We replicated and extended the United States investigators' approach using contemporary, population‐based data in Ontario, Canada. In Ontario, the risk of RSV hospitalisation is generally greatest at the lowest chronological ages [33] and the risk of RSV hospitalisation may be greater during more intense RSV seasons (characterised by more cases/hospitalisations). These characteristics are a function of infants' calendar times at birth, which are not thought to be influenced by individual‐level (e.g., genetic) common causes of RSV LRTI and asthma. We therefore explored potential causality of the relationship between RSV and paediatric asthma by exploiting ‘quasi‐random’ variation with respect to individual‐level risk factors for severe RSV infection. We first replicated the United States investigators' approach by examining the relationship between each infant's calendar time at birth relative to their first RSV season and asthma at 5 years of age (objective 1). We further investigated this relationship by examining the specific timing and magnitude of RSV seasons from year to year and the probability of asthma at 5 years of age (objective 2).

## 2 Methods

### 2.1 Design, Population and Data Sources

Ontario has 16.3 million residents comprising ~40% of Canada's population [34]. In Ontario, there are approximately 145,000 annual births and 97% of them occur in hospitals [35]. We included all hospital‐based live births from April 1, 2002 to March 30, 2013, which enabled a 5‐year follow‐up period. We used data from the Registered Persons, Ontario Asthma, Ontario Health Insurance Plan, Canadian Institute for Health Information Discharge Abstract, and MOMBABY administrative databases housed at ICES Ontario (https://www.ices.on.ca/; Appendix 1, Figure S1 and Table S1) [36]. These datasets were linked using unique encoded identifiers and analysed at ICES. Infant records with missing/invalid ICES Key Numbers/dates of birth, missing infant sex, and/or linkage warnings were excluded. Infants who died or migrated within the first 5 years of life were excluded from some primary analyses.

### 2.2 Measures

#### 2.2.1 Exposures

We explored calendar time at birth as a source of ‘quasi‐random’ variation with respect to individual‐level risk factors for severe RSV infection (Figure S2, panel A) [37, 38]. For objective 1, we used an individual‐level exposure: infant's age (in weeks) during the week when the RSV peak occurred in their first year of life (hereafter, ‘infant age at RSV peak’). Each week spanned Sunday–Saturday and each season spanned September 1–August 31; RSV peak was identified as the week with the highest number of hospitalised RSV cases among infants aged < 1 year (Table S2). RSV hospitalisations were identified using International Classification of Diseases 10th Revision (ICD‐10‐CA) codes: J12.1, J20.5, J21.0, and/or B97.4 [39]. Sensitivity and specificity of these codes for identifying RSV in Ontario administrative data are 98% and > 99%, respectively [39]. Infant age at RSV peak was calculated by subtracting each infant's week of birth from the week of each infant's first RSV peak. For objective 2, we used a calendar week‐level exposure: the weekly incidence of RSV hospitalisation among infants aged < 1 year (hereafter, ‘weekly incidence of RSV hospitalisation’; Appendix 2), which served as a proxy for the timing and magnitude of RSV seasons.

We similarly examined influenza and rotavirus. Influenza often co‐circulates with RSV [40], can cause severe LRTI, and was included in the winter virus definition employed by Wu and colleagues [30, 41]. We identified influenza hospitalisation among infants aged < 1 year using ICD‐10‐CA codes (J09, J10.0, J10.1, J10.8, J11.0, J11.1, J11.8) [42]. Rotavirus (a seasonal gastrointestinal virus) was incorporated as a negative control exposure [43], which is thought to be susceptible to similar biases (including unmeasured confounding) as those in an exposure‐outcome relationship of interest, but without a causal effect on the outcome [43]. We identified rotavirus/gastroenteritis hospitalisation among infants aged < 1 year using ICD‐10‐CA codes (A08.0, A08.3, A08.4, A08.5, A09, K52.9) [44]. We calculated infant ages at the influenza and rotavirus peaks and the weekly incidences of influenza and rotavirus hospitalisations, as described above.

#### 2.2.2 Outcomes

The outcome for objective 1 was asthma at 5 years of age (hereafter ‘paediatric asthma’), ascertained by presence in the Ontario Asthma Dataset [45, 46, 47, 48]. The sensitivity and specificity of the case‐finding algorithm are 81% and 90%, respectively, among children aged 1–5 years, and 81%–84% and 77%–81%, respectively, among adults [45, 48]. We considered those with asthma < 3 years of age as cases in primary analysis only if ≥ 1 additional inpatient/outpatient visit for asthma was recorded from 3–5 years of age. We imposed this adaptation because asthma diagnostic tests are only recommended at ≥ 3 years of age [49]. The outcome for objective 2 was the number of infants with asthma at 5 years of age divided by the number of infants born in each calendar week (hereafter ‘probability of paediatric asthma’).

#### 2.2.3 Covariates

We considered infant's sex, virus season of birth, singleton/multiple pregnancy, and pregnant person's age at delivery, number of previous live births, and asthma as potential confounders for objective 1. We expected that adjustment for these individual‐level characteristics would have little impact on effect estimates, given that they are likely independent of calendar time at birth.

### 2.3 Statistical Analysis

For objective 1, we plotted crude odds and LOESS‐smoothed curves of paediatric asthma by infant age at RSV peak. We then used logistic regression to estimate and plot the unadjusted (univariable model) and adjusted (multivariable model) predicted odds ratio of paediatric asthma by infant age at RSV peak (referent = 0 weeks of age; Appendix 3, Equation 1). We conceptualised infant age at the RSV peak using restricted cubic splines with 4 knots at quantiles of the data and used generalised estimating equations with unstructured covariance to account for clustering of multiple infants per mother. We visually identified infant ages associated with the highest odds and predicted odds ratios for paediatric asthma. We repeated these analyses for influenza and rotavirus, and conducted these analyses separately for RSV, influenza, and rotavirus since infant ages at viral peaks are temporally related.

For objective 2, we plotted weekly incidences of RSV, influenza, and rotavirus hospitalisations by: (a) the calendar week at which they occurred; and (b) offset by infant ages at RSV, influenza, and rotavirus peaks that conferred maximum crude odds of paediatric asthma (ascertained from objective 1). We used linear regression to quantify the relationship between the probability of paediatric asthma and the weekly incidence of RSV, influenza, and rotavirus hospitalisations (Appendix 3, Equation 2). We performed these analyses to determine whether the specific timing and magnitude of viral seasons were associated with higher or lower probabilities of paediatric asthma among infants born during a given calendar week.

#### 2.3.1 Sensitivity Analysis

For objective 1, we varied the outcome definition and model specification and explored the role of gestational age at birth, which may lie on the causal pathway (see Appendix 4 for additional details on the sensitivity analyses for objective 1). For objective 2, we varied the exposure definition by using infant ages at viruses' peaks that conferred the maximum predicted unadjusted and adjusted odds ratios of paediatric asthma. We additionally adjusted for probabilities of preterm birth as well as probabilities of RSV, influenza, and rotavirus hospitalisation in the first year of life, which is a different quantity than the weekly incidence of virus‐related hospitalisation (our proxy for virus season's timing and magnitude; Appendix 2) to provide a crude assessment of mediation by these factors [50].

Datasets were compiled using SAS Enterprise Guide version 8.3 update 7 (8.3.7.202; SAS Institute, North Carolina) and analyses were conducted using Stata version 15.1 (StataCorp, Texas).

### 2.4 Missing Data

There were < 5% of missing data for covariates. Objective 1 included infants with < 5 years of follow‐up due to death or migration (2.2%) in crude/descriptive analyses (Figures 1 and 2) and excluded these infants in unadjusted/adjusted analyses (Figure 3). Objective 2 included infants with < 5 years of follow‐up due to death or migration in crude/descriptive analyses (Figure 4) and regression analyses. Objective 2 regression analyses included viral seasons from September 2002–August 2013. Numerators for weekly incidence of virus‐related hospitalisations and probability of virus‐related hospitalisations were set to 6 if < 6 to suppress small cells and denominators were set to the mean number of infants born per week for < 1% of calendar weeks with missing values. Objective 2 regression models that incorporated offset terms excluded between 11 and 19 weeks (< 4%) corresponding to the maximum offset term used in each model.

**FIGURE 1:**
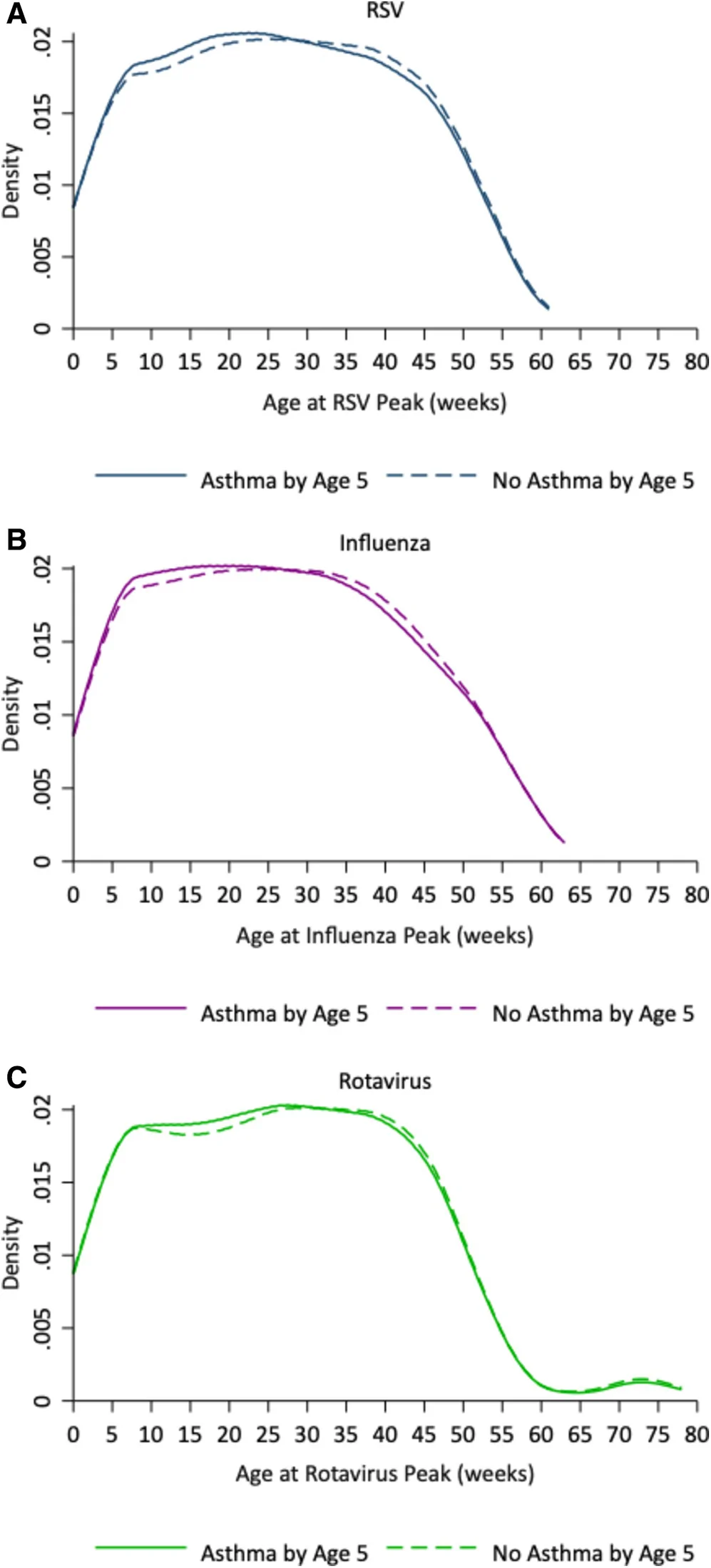
Distribution of infant age at first RSV (Panel A), influenza (Panel B), and rotavirus (Panel C) peaks by asthma/no asthma at 5 years of age. The *y*‐axis denotes the density of observations by infant age at viral peak.

**FIGURE 2:**
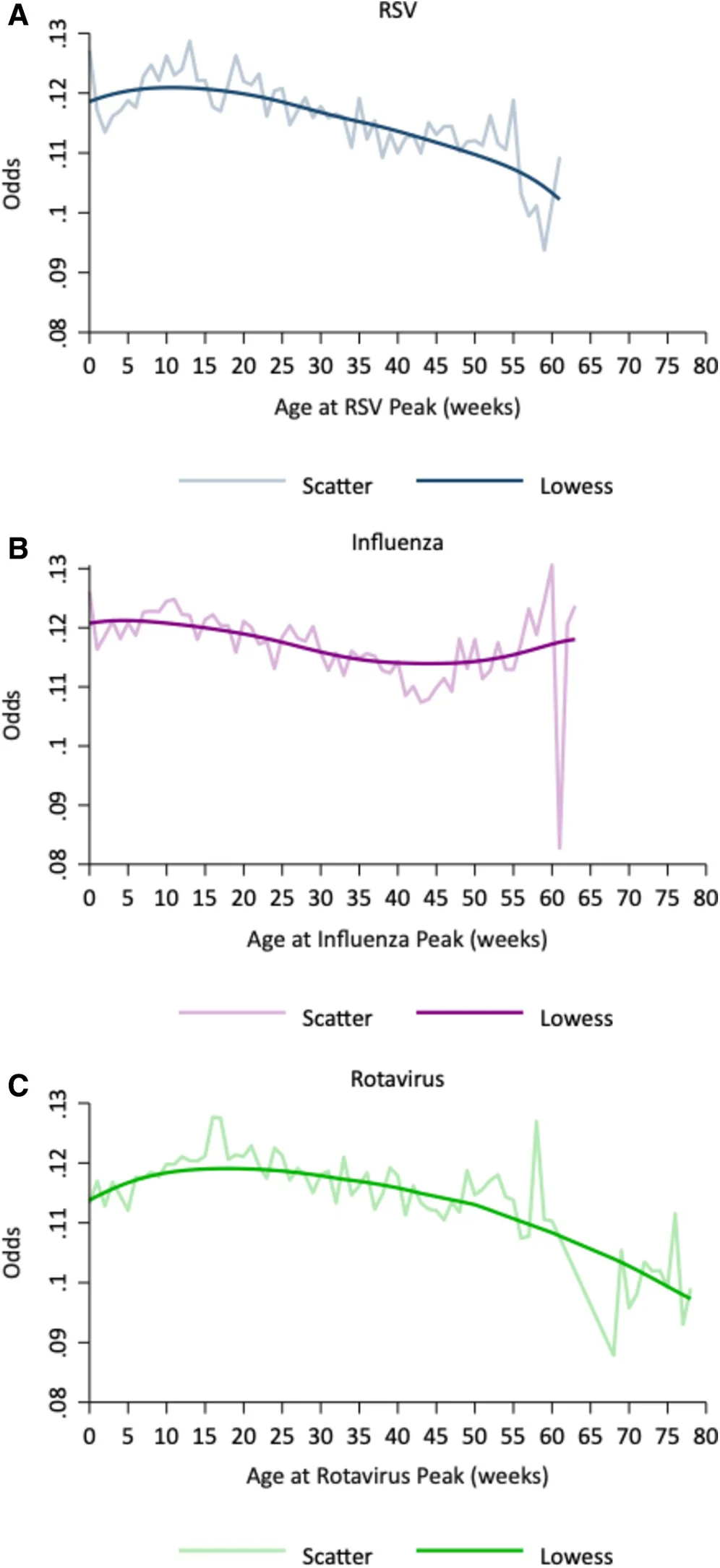
Crude odds of asthma at 5 years of age by infant age at first RSV (Panel A), influenza (Panel B), and rotavirus (Panel C) peaks.

**FIGURE 3:**
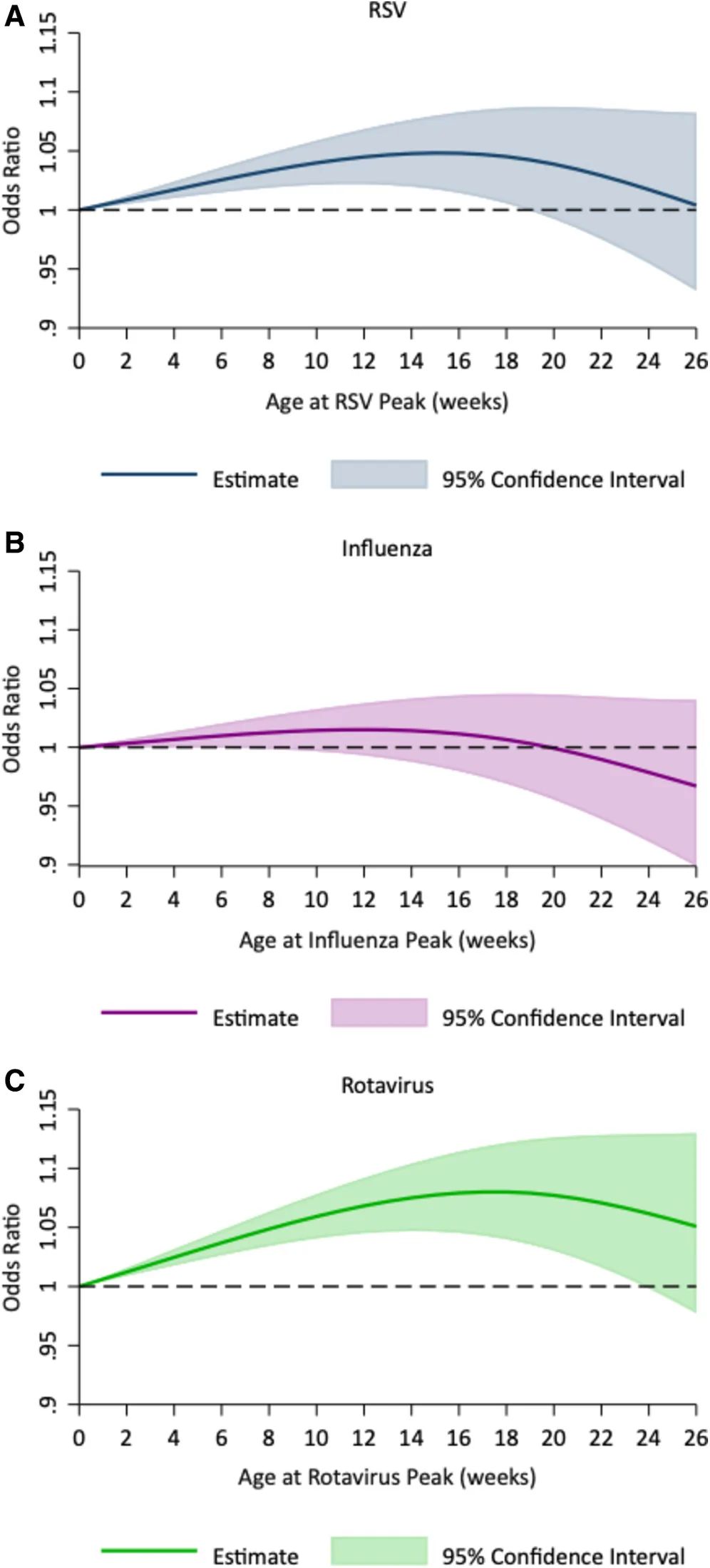
Adjusted predicted odds ratio of asthma at 5 years of age by infant age at first RSV (Panel A), influenza (Panel B), and rotavirus (Panel C) peaks. Predicted odds ratios are adjusted for infant sex, virus season of birth, and pregnant persons' age, previous live births, and asthma. Infant ages from 0 to 26 weeks displayed.

**FIGURE 4:**
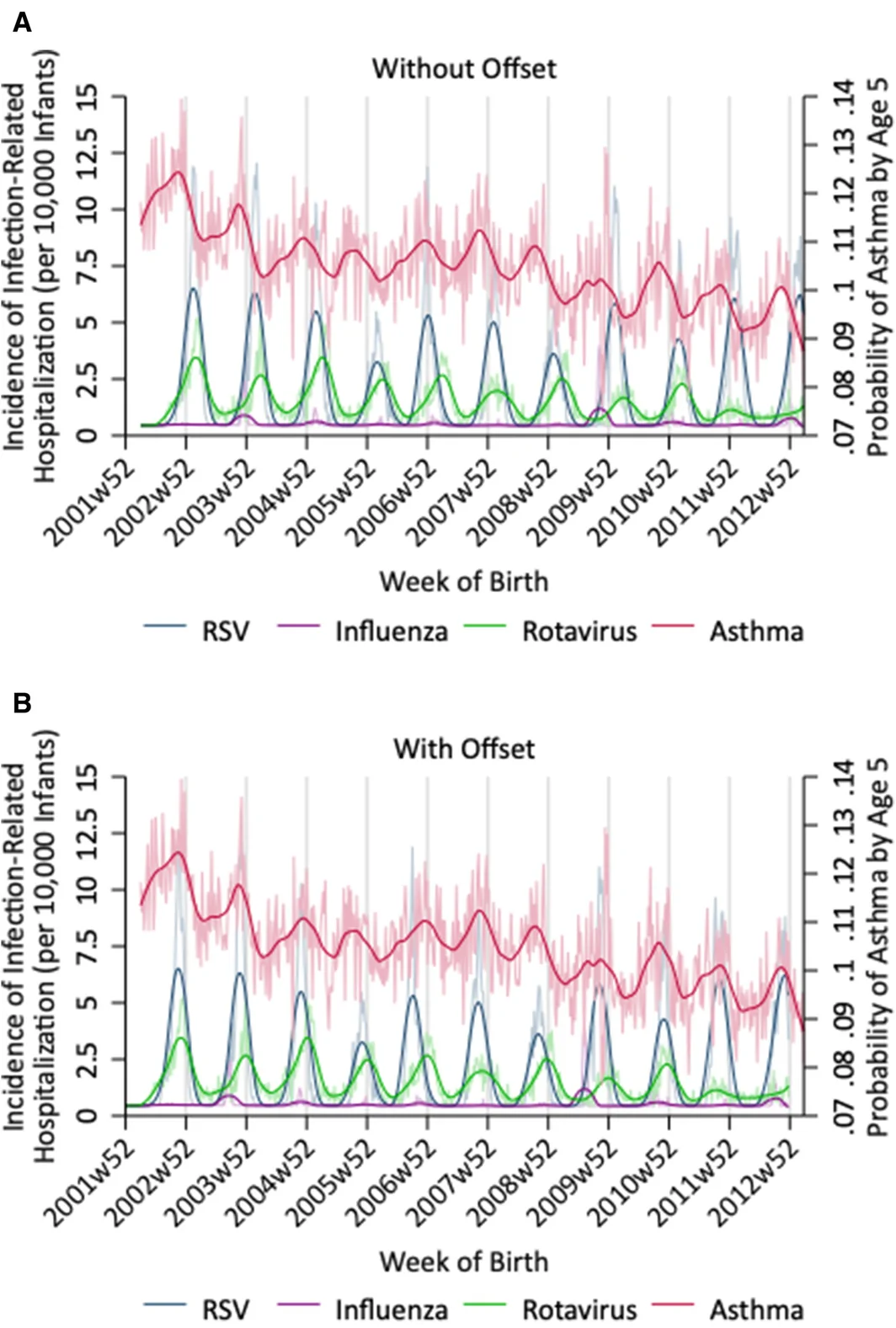
Incidence of virus‐related hospitalisations by calendar week and probability of asthma at 5 years of age by calendar week of birth. Incidence of virus‐related hospitalisations by calendar week plotted without offset (Panel A) and with offset (Panel B) at the age at which the crude log‐odds of asthma at age 5 were highest. The offset terms for Panel B were 13 weeks for RSV, 11 weeks for influenza, and 16 weeks for rotavirus. To facilitate visualisation in figures, incidence of influenza hospitalisation was set to a maximum of 12.5 per 10,000 infants if greater than this value (i.e., during the 2009–2010 A/H1N1 influenza pandemic).

### 2.5 Ethics Approval

This study received Research Ethics Board (REB) approval from the Children's Hospital of Eastern Ontario Research Institute (REB Protocol No: 19/12PE).

## 3 Results

We analysed 1,437,731 infants (Table 1). Distributions of most baseline characteristics were similar across infant age at RSV peak quartiles, although the percentage of preterm infants was slightly higher in the first quartile (8.3% vs. 7.9% overall).

**TABLE 1:** Pregnant person, infant and pregnancy characteristics by quartile of infant age at first respiratory syncytial virus (RSV) peak.

	Infant age at RSV peak				
	Quartile 1 (*n* = 364,515)	Quartile 2 (*n* = 373,623)	Quartile 3 (*n* = 341,724)	Quartile 4 (*n* = 357,869)	All (*n* = 1,437,731)
Infant age at RSV peak (weeks)^a^	0, 14	15, 27	28, 39	40, 61	0, 61
Infant age at RSV peak (weeks)	7 (4, 11)	21 (18, 24)	33 (30, 36)	46 (43, 50)	27 (14, 39)
Infant age at Influenza peak (weeks)	12 (6, 46)	19 (13, 25)	32 (25, 37)	41 (33, 47)	26 (14, 39)
Infant age at Rotavirus peak (weeks)	13 (8; 18)	25 (20; 30)	37 (33; 42)	41 (6; 48)	27 (14; 39)
Missing	9950 (2.7)	0 (0.00)	0 (0.00)	0 (0.00)	9950 (0.7)
Infant sex					
Female	177,792 (48.8)	182,458 (48.8)	165,875 (48.5)	174,432 (48.7)	700,557 (48.7)
Male	186,709 (51.2)	191,150 (51.2)	175,830 (51.5)	183,424 (51.3)	737,113 (51.3)
Other	14 (0.00)	15 (0.00)	19 (0.01)	13 (0.00)	61 (0.00)
Pregnant person age (years)^b^	29.99 (5.57)	29.95 (5.50)	29.94 (5.46)	29.94 (5.52)	29.96 (5.51)
Plurality					
Singleton	351,551 (96.4)	361,254 (96.7)	329,937 (96.6)	345,185 (96.5)	1,387,927 (96.5)
Multiple	12,964 (3.6)	12,369 (3.3)	11,787 (3.4)	12,684 (3.5)	49,804 (3.5)
Number of previous live births					
0	164,661 (45.2)	166,621 (44.6)	151,709 (44.4)	158,930 (44.4)	641,921 (44.6)
1	129,780 (35.6)	134,553 (36.0)	124,371 (36.4)	130,416 (36.4)	519,120 (36.1)
≥ 2	70,074 (19.2)	72,449 (19.4)	65,644 (19.2)	68,523 (19.1)	276,690 (19.2)
Pregnant person asthma					
No	316,978 (87.0)	324,982 (87.0)	297,676 (87.1)	310,496 (86.8)	1,250,132 (87.0)
Yes	47,537 (13.0)	48,641 (13.0)	44,048 (12.9)	47,373 (13.2)	187,599 (13.0)
Gestational age at delivery (weeks)	39 (38; 40)	39 (38; 40)	39 (38; 40)	39 (38; 40)	39 (38; 40)
Missing	57 (0.02)	61 (0.02)	58 (0.02)	63 (0.02)	239 (0.02)
Preterm birth					
No (≥ 37 weeks at birth)	334,350 (91.7)	345,406 (92.4)	314,542 (92.0)	329,106 (92.0)	1,323,404 (92.0)
Yes (< 37 weeks at birth)	30,108 (8.3)	28,156 (7.5)	27,124 (7.9)	28,700 (8.0)	114,088 (7.9)
Missing	57 (0.02)	61 (0.02)	58 (0.02)	63 (0.02)	239 (0.02)
Calendar year of birth	2007 (2005, 2010)	2007 (2004,2010)	2007 (2004, 2010)	2008 (2005, 2010)	2007 (2005, 2010)
RSV hospitalisation by 1 year					
No	358,411 (98.3)	369,984 (99.0)	339,880 (99.5)	355,723 (99.4)	1,423,998 (99.0)
Yes	6104 (1.7)	3639 (1.0)	1844 (0.5)	2146 (0.6)	13,733 (1.0)
Influenza hospitalisation by 1 year					
No	364,108 (99.9)	373,245 (99.9)	341,478 (99.9)	357,546 (99.9)	1,436,377 (99.9)
Yes	407 (0.1)	378 (0.1)	246 (0.1)	323 (0.1)	1354 (0.1)
Rotavirus hospitalisation by 1 year					
No	362,295 (99.4)	371,136 (99.3)	339,100 (99.2)	355,378 (99.3)	1,427,909 (99.3)
Yes	2220 (0.6)	2487 (0.7)	2624 (0.8)	2491 (0.7)	9822 (0.7)
Asthma at 5 years with 3–5 year criteria (primary definition)					
No	325,179 (89.2)	333,587 (89.3)	306,439 (89.7)	321,842 (89.9)	1,287,047 (89.5)
Yes	39,336 (10.8)	40,036 (10.7)	35,285 (10.3)	36,027 (10.1)	150,684 (10.5)
Asthma at 5 years without 3–5 year criteria (secondary definition)					
No	321,213 (88.1)	329,489 (88.2)	302,432 (88.5)	318,080 (88.9)	1,271,214 (88.4)
Yes	43,302 (11.9)	44,134 (11.8)	39,292 (11.5)	39,789 (11.1)	166,517 (11.6)
Asthma at 3 years (secondary definition)					
No	306,301 (84.0)	314,215 (84.1)	289,095 (84.6)	304,426 (85.1)	1,214,037 (84.4)
Yes	58,214 (16.0)	59,408 (15.9)	52,629 (15.4)	53,443 (14.9)	223,694 (15.6)
Asthma at 6 years (secondary definition)					
No	281,622 (77.3)	290,299 (77.7)	265,960 (77.8)	280,327 (77.3)	1,118,208 (77.8)
Yes	41,730 (11.4)	42,575 (11.4)	37,670 (11.0)	38,983 (10.9)	160,958 (11.2)
N/A^c^	41,163 (11.3)	40,749 (10.9)	38,094 (11.1)	38,559 (10.8)	158,565 (11.0)
Asthma at 8 years (secondary definition)					
No	215,290 (59.1)	224,437 (60.1)	206,427 (60.4)	215,849 (60.3)	862,003 (60.0)
Yes	38,925 (10.7)	40,101 (10.7)	36,148 (10.6)	37,187 (10.4)	152,361 (10.6)
N/A^d^	110,300 (30.3)	109,085 (29.2)	99,149 (29.0)	104,833 (29.3)	423,367 (29.4)

*Note:* Values are expressed as a number (percentage) for categorical variables and median (25th percentile, 75th percentile) for continuous variables unless otherwise stated.

^a^ Values expressed as minimum; maximum.

^b^ Values expressed as mean (standard deviation).

^c^ N/A represents children with less than 6 years of follow‐up.

^d^ N/A represents children with less than 8 years of follow‐up.

Overall, 1.0%, 0.1%, and 0.7% of infants were hospitalised with RSV, influenza, and rotavirus, respectively. Hospitalisation with RSV and influenza was most frequent in the first quartile (1.7% and 0.1%, respectively), while the percentage of infants hospitalised for rotavirus increased with higher quartiles. Overall, 10.5% of children met the criteria for our primary definition of paediatric asthma.

### 3.1 Objective 1

The distribution of infant age at RSV peak was lower among those who had (versus did not have) paediatric asthma (Figure 1). The highest crude odds of paediatric asthma were observed among infants aged 13 weeks at RSV peak (Figure 2, light line), while the LOESS‐smoothed curve suggested a slightly broader range (Figure 2, bold line). Primary analyses used offsets of 13, 11, and 16 weeks for RSV, influenza, and rotavirus, respectively, based on the week of age at which the crude log‐odds of paediatric asthma were highest (Figure 2).

The unadjusted and adjusted predicted odds ratios of paediatric asthma were highest at infant ages of approximately 15–16 weeks at RSV, 12–13 weeks at influenza, and 17–19 weeks at rotavirus peaks (Figure S3 and Figure 3 present predicted unadjusted and adjusted odds ratios, respectively, across infant ages of 0–26 weeks; Figures S4 and S5 extend across 0–52 weeks). Adjustment for infant's sex and virus season of birth, pregnant person's age (see Figure S6 for the non‐linear relationship estimated by restricted cubic splines), asthma, and previous live births had a negligible impact on inferences.

Inferences were largely unchanged in sensitivity analyses that conceptualised paediatric asthma using alternative definitions, incorporated infants with < 5 years of follow‐up due to death or migration, or stratified by infant sex (male/female) (Table S3, models 4–6, 8–11 and 15–16). Adjustment for characteristics that may be on the causal pathway (specifically gestational age at birth—see Figure S7 for non‐linear relationship estimated by restricted cubic splines—and RSV hospitalisation) did not change inferences (Table S3, models 12–14 and 17).

### 3.2 Objective 2

Weekly incidences of RSV and rotavirus hospitalisations exhibited a visually sinusoidal (or seasonal) pattern (Figure 4). RSV and rotavirus season's peaks varied in height across the study period. Influenza season was less evident (except during the 2009–2010 A/H1N1 influenza pandemic) because influenza hospitalisation among infants was uncommon.

The probability of paediatric asthma decreased over time from approximately 12% to 9% during the study period. As with RSV and rotavirus seasons, the probability of paediatric asthma followed a visually sinusoidal pattern, with peaks that appeared shorter and wider than those of RSV seasons. Coefficients that quantified the relationship between weekly incidences of RSV, influenza, and rotavirus hospitalisation (offset by infant ages at viral peaks that conferred the highest crude odds of paediatric asthma) and probability of paediatric asthma are displayed in Table 2 and were interpreted by comparing their magnitude and precision across viruses. Coefficients represented the percentage‐point change in probability of paediatric asthma per percentage‐point change in weekly incidence of virus‐related hospitalisation. For example, in the main model, the coefficient for RSV was 3.15 (95% confidence interval [CI] −0.07, 6.36), indicating that for every 1 percentage‐point increase in weekly incidence of RSV hospitalisations (offset by 13 weeks), there was a 3.15 percentage‐point increase in the probability of paediatric asthma among those born in the corresponding calendar week. The coefficient for influenza was 19.71 (95% CI −4.04, 43.46); its magnitude varied in sensitivity analyses, and its precision remained low, likely due to the small number of influenza hospitalisations among infants. Conversely, the coefficient for rotavirus was 45.50 (95% CI 36.06, 54.94); its magnitude and precision were similar across most sensitivity analyses. The magnitudes of the RSV and influenza coefficients were highly sensitive to the choice of offset term (Table 2).

**TABLE 2:** Results from population‐level regression models (objective 2) where the dependent variable was the weekly probability of asthma at 5 years of age, and the independent variables were the weekly incidence of RSV, influenza, and rotavirus hospitalisations among infants < 1 year of age.

Objective 2 regression model	*N* weeks	RSV lag (weeks)	RSV coefficient	Influenza lag (weeks)	Influenza coefficient	Rotavirus lag (weeks)	Rotavirus coefficient
No offset	552	0	−4.19 (−7.57, −0.81)	0	11.15 (−16.28, 38.58)	0	11.91 (1.97, 21.86)
Offset from crude odds^a^	536	13	3.15 (−0.07, 6.36)	11	19.71 (−4.04, 43.46)	16	45.50 (36.06, 54.94)
Offset from univariable models^b^	533	16	0.09 (−3.14, 3.33)	13	1.71 (−22.19, 25.62)	19	51.32 (41.82, 60.83)
Offset from multivariable models^c^	535	15	1.86 (−1.30, 5.01)	12	11.94 (−12.05, 35.93)	17	47.37 (38.10, 56.63)

^a^ Offset term derived from the infant age associated with highest crude odds of asthma from model in Figure 2.

^b^ Offset term derived from the infant age associated with highest odds of asthma from univariable models in Figure S3.

^c^ Offset term derived from the infant age associated with highest odds of asthma from multivariable models in Figure 3.

The magnitudes of RSV and influenza coefficients were also sensitive to whether weekly incidences of virus‐related hospitalisations were examined separately or concurrently (Table S4), although their direction and relative magnitudes were similar. The magnitude of the rotavirus coefficient was largely unchanged throughout sensitivity analyses but decreased to 19.92 (95% CI 10.99–28.84) when a linear term for year was incorporated (Table S4). Inferences were similar when incorporating the probability of preterm birth (Figure S8).

Given the unexpected and persistent observed relationship between the timing and magnitude of rotavirus seasons and the probability of paediatric asthma, we further assessed whether the calendar week of birth was a suitable source of ‘quasi‐random’ variation for severe RSV, influenza, and rotavirus infection. For example, if rotavirus season's timing and magnitude was a suitable source of ‘quasi‐random’ variation in this context, we would expect its relationship with asthma to be mediated by severe rotavirus infection. Figures S9–S11 display the weekly incidence of virus‐related hospitalisations alongside the probability of a virus‐related hospitalisation in the first year of life for RSV, influenza, and rotavirus, respectively. The peaks and valleys of these two quantities closely follow each other for RSV and influenza (Figures S9 and S10), but not for rotavirus (Figure S11). Additionally, the coefficient for RSV lessened in magnitude from 12.01 (95% CI 9.17, 14.84; Table S4, model 10) to 7.81 (95% CI 4.29, 11.329; Table S4, model 15) with adjustment for probability of RSV hospitalisation in infancy, while the coefficient for rotavirus remained similar with/without adjustment for probability of rotavirus hospitalisation in infancy (Table S4, models 14 and 17). These analyses indicate that calendar time at birth may have been a suitable source of ‘quasi‐random’ variation for severe RSV infection, but not for severe rotavirus infection.

## 4 Comment

### 4.1 Principal Findings

We found some evidence for a small role of RSV season's timing and magnitude in causing paediatric asthma, robust to sensitivity analyses and appearing partially mediated by RSV hospitalisation, though this relationship may be due to shared seasonal causes. An unexpected relationship between rotavirus seasonality and asthma emerged but was sensitive to model parameters and, importantly, did not appear to be mediated by rotavirus hospitalisation. We observed few influenza hospitalisations among infants, which precluded any strong inferences about influenza seasonality. Taken together, these findings suggest that other mechanisms, including RSV infection without severe LRTI (Figure S2, panel B) and seasonal factors (Figure S2, panel C), may contribute to observed associations between RSV infection and asthma.

### 4.2 Strengths of the Study

Strengths of our study include its population‐based design and use of calendar time at birth (including yearly variation in virus seasons' magnitudes) as a source of ‘quasi‐random’ variation for RSV infection (and subsequent LRTI), which is less susceptible to confounding by individual‐level characteristics [30]. Our intended use of rotavirus as a negative control detected possible confounding of calendar time at birth and paediatric asthma by seasonal factors. Additionally, administrative codes for RSV, influenza‐, and rotavirus‐related hospitalisations and the Ontario Asthma Dataset were previously validated in our study population [39, 42, 44, 45, 46, 47, 48].

### 4.3 Limitations of the Data

Our study had several limitations. We used the incidence of virus‐related hospitalisations among infants instead of weekly percentage positive specimens in the general population (which were unavailable in our study). Testing practices (and identification of virus‐related hospitalisations in administrative data) may have varied over the study period. Influenza and rotavirus vaccines were available (with varying levels of uptake and coverage) during the study period [51, 52]. However, this does not affect inferences since we do not expect vaccine receipt to be associated with paediatric asthma except through its effect on severe viral infection, and vaccine receipt is on the causal pathway between calendar time at birth and severe viral infection (Figure S2, panel D). We were unable to examine the role of other respiratory viruses, such as human metapneumovirus; however, this would only confound our findings if the relative magnitude of its seasons approximated that of either RSV or rotavirus.

### 4.4 Interpretation

Our finding that infants aged 13–16 weeks at RSV peak had the highest odds of paediatric asthma mirrors results from an earlier study, which found that the risk of asthma was highest among infants aged 121 days (~17 weeks) at winter virus peak (adjusted odds ratio of 1.29, 95% CI 1.19, 1.40; Table S5) [30]. This point estimate is larger in magnitude than ours, which approached 1.05 (Figure 3 and Figure S3). We used birth during the week of RSV peak as our referent (instead of 365 days after the winter virus peak), and our point estimate was 0.9–1 in the latter half of the range of infant age at RSV peak (Figures S4 and S5); this is consistent with the earlier study reporting higher baseline odds for asthma at 0 versus 52 weeks.

While our results are not directly comparable to previous studies that support a causal relationship between severe RSV infection and asthma (due to differences in exposures and outcomes), our point estimates for paediatric asthma by infant age at RSV peak are generally smaller in magnitude [12, 13, 22, 23, 24, 25, 26, 27, 29]. Given that the RSV peak typically occurs in February (Table S2), our findings are generally consistent with previous population‐based studies that report a higher risk of asthma among infants born in autumn [31, 32].

The observed correlation between the timing and magnitude of rotavirus seasons and subsequent asthma should be interpreted with caution, as our study was not designed to assess the impact of rotavirus seasonality. A recent cohort study in Denmark found an association between infant gut virome and the risk of paediatric asthma, which may support the biological plausibility of this relationship [53]; however, this study did not specifically examine rotavirus. Our observed correlation is more compatible with unmeasured confounding by individual‐level and/or seasonal characteristics; environmental and/or seasonal factors could worsen rotavirus seasons and increase susceptibility to childhood asthma. For example, some studies suggest that cold temperature and low humidity are risk factors for more severe rotavirus seasons [54, 55]. Children may spend more time indoors during colder winters, which could increase the risk of asthma [56]. Potential unmeasured confounding by seasonal characteristics highlights the challenge of using calendar time at birth as a source of quasi‐random variation.

## 5 Conclusions

We found limited evidence for a small causal effect of severe RSV infection on paediatric asthma, yet we could not rule out the contribution of other seasonal factors. Exploiting regional variation in typical length and timing of the RSV season, and in population‐level RSV preventive measures, may further contribute to our understanding of this relationship.

## Author Contributions

M.C.D., D.R.F., and D.B.F. conceptualised and designed the study. M.C.D. and S.E.S. conducted the data analysis. M.C.D. and D.B.F. interpreted the findings and drafted the manuscript. All authors contributed to the interpretation of the findings, the critical review, and the revision of the final manuscript. All authors approve the final manuscript as submitted. M.C.D. will act as guarantor for the paper.

## Funding

This work was supported by the World Health Organisation (WHO) through a grant from the Bill and Melinda Gates Foundation (Global Health Grant OPP1114766); and ICES, which is funded by an annual grant from the Ontario Ministry of Health (MOH) and the Ministry of Long‐Term Care (MLTC). JCK is supported by a Clinician Scientist Award from the University of Toronto Department of Family and Community Medicine.

## Conflicts of Interest

During the conduct of this work, D.B.F. worked for the University of Ottawa and had academic appointments at the Children's Hospital of Eastern Ontario Research Institute and ICES; she is currently employed by Pfizer. The other authors declare no conflicts of interest.

## Supporting information


**Appendix 1.** Description of study datasets.
**Figure S1:** Relationship/linkage between study datasets.
**Table S1:** Summary of study datasets.
**Figure S2:** Causal diagrams for the source of quasi‐random variation presented throughout our manuscript (Panel A), as well as the potential roles of RSV infection in the absence of severe RSV LRTI (Panel B), seasonal factors (Panel C), and RSV vaccine (Panel D).
**Table S2:** Calendar weeks during which the highest number of RSV, influenza, and rotavirus hospitalisations occurred (i.e., definition of viral peaks).
**Appendix 2**. Formulae for incidence of virus‐related hospitalisations (i.e., proxy for seasonality) versus probability of virus‐related hospitalisation.
**Appendix 3**. Equations for regression analyses for objectives 1 and 2.
**Appendix 4**. Additional details about sensitivity analyses for Objective 1.
**Figure S3:** Unadjusted predicted odds ratio of asthma at 5 years of age by infant age at first RSV (Panel A), influenza (Panel B), and rotavirus (Panel C) peaks. Infant ages from 0 to 26 weeks are displayed and infants with < 5 years of follow‐up were excluded.
**Figure S4:** Unadjusted predicted odds ratio of asthma at 5 years of age by infant age at first RSV (panel A), influenza (Panel B), and rotavirus (Panel C) peaks. Infant ages from 0 to 52 weeks are displayed and infants with < 5 years of follow‐up were excluded.
**Figure S5:** Adjusted predicted odds ratio of asthma at 5 years of age by infant age at first RSV (Panel A), influenza (Panel B), and rotavirus (Panel C) peaks. Predicted odds ratios are adjusted for infant sex, pregnant person' age, pregnant person asthma, previous live births, and year of birth. Infant ages from 0 to 52 weeks are displayed and infants with < 5 years of follow‐up were excluded.
**Figure S6:** Predicted odds ratio of asthma at 5 years of age by pregnant person age modelled using splines (referent = youngest age). Infants with < 5 years of follow‐up were excluded.
**Table S3:** Results from individual‐level sensitivity analyses where the dependent variable was asthma at 5 years of age.
**Figure S7:** Predicted odds ratio of asthma at 5 years of age by gestational age at birth modelled using splines (referent = youngest age). Infants with < 5 years of follow‐up and/or missing gestational age at birth were excluded.
**Table S4:** Results from population‐level sensitivity analyses where the dependent variable is probability of asthma at 5 years of age as a percentage of infants born in each calendar week.
**Figure S8:** Incidence of infection‐related hospitalisations by calendar week and probability of preterm birth and asthma at 5 years of age by calendar week of birth. Incidence of infection‐related hospitalisations by calendar week plotted without offset (Panel A) and with offset (Panel B) by age at which crude log‐odds of asthma at age 5 was highest. The offset terms for Panel B were 13 weeks for RSV, 11 weeks for influenza, and 16 weeks for rotavirus. To facilitate visualisation in figures, incidence of influenza‐related hospitalisations was set to a maximum of 12.5 per 10,000 infants if greater than this value (i.e., during the 2009‐2010 A/H1N1 influenza pandemic).
**Figure S9:** Incidence of RSV hospitalisation among infants by calendar week plotted alongside probability of RSV hospitalisation in the first year of life by week of birth (see Appendix 2 for definitions). Incidence of RSV hospitalisations by calendar week plotted without offset (Panel A) and with offset (Panel B) by age at which crude log‐odds of asthma at age 5 was highest (i.e., 13 weeks).
**Figure S10:** Incidence of influenza hospitalisation among infants by calendar week plotted alongside probability of influenza hospitalisation in the first year of life by week of birth (see Appendix 2 for definitions). Incidence of influenza hospitalisations by calendar week plotted without offset (Panel A) and with offset (Panel B) by age at which crude log‐odds of asthma at age 5 was highest (i.e., 11 weeks). To facilitate visualisation in figures, incidence of influenza hospitalisation was set to a maximum of 12.5 per 10,000 infants if greater than this value and probability of influenza hospitalisation was set to a maximum of 0.008 if greater than this value (i.e., during the 2009–2010 A/H1N1 influenza pandemic).
**Figure S11:** Incidence of rotavirus hospitalisation among infants by calendar week plotted alongside probability of rotavirus hospitalisation in the first year of life by week of birth (see Appendix 2 for definitions). Incidence of rotavirus hospitalisations by calendar week plotted without offset (Panel A) and with offset (Panel B) by age at which crude log‐odds of asthma at age 5 was highest (i.e., 16 weeks).
**Table S5:** Comparison of RSV measures and study population between Wu et al. and current study.

## Data Availability

The dataset from this study is held securely in coded form at ICES. While legal data sharing agreements between ICES and data providers (e.g., healthcare organisations and government) prohibit ICES from making the dataset publicly available, access may be granted to those who meet pre‐specified criteria for confidential access, available at www.ices.on.ca/DAS (email: das@ices.on.ca). The full dataset creation plan and underlying analytic code are available from authors upon request, understanding that the computer programmes may rely upon coding templates or macros that are unique to ICES and are therefore either inaccessible or may require modification. ICES is a prescribed entity under Ontario's Personal Health Information Protection Act (PHIPA). Section 45 of PHIPA authorises ICES to collect personal health information, without consent, for the purpose of analysis or compiling statistical information with respect to the management of, evaluation or monitoring of, the allocation of resources to or planning for all or part of the health system. The use of the data in this project is authorised under section 45 and approved by ICES' Privacy and Legal Office.

## References

[1] W. P. Glezen , “Risk of Primary Infection and Reinfection With Respiratory Syncytial Virus,” Archives of Pediatrics & Adolescent Medicine 140 (1986): 543. 10.1001/archpedi.1986.02140200053026 3706232

[2] Y. Li , X. Wang , D. M. Blau , et al., “Global, Regional, and National Disease Burden Estimates of Acute Lower Respiratory Infections due to Respiratory Syncytial Virus in Children Younger Than 5 Years in 2019: A Systematic Analysis,” Lancet 399 (2022): 2047–2064. 10.1016/S0140-6736(22)00478-0 PMC7613574 35598608

[3] R. C. Welliver , P. A. Checchia , J. H. Bauman , A. W. Fernandes , P. J. Mahadevia , and C. B. Hall , “Fatality Rates in Published Reports of RSV Hospitalizations Among High‐Risk and Otherwise Healthy Children,” Current Medical Research and Opinion 26 (2010): 2175–2181. 10.1185/03007995.2010.505126 20666690

[4] T. Shi , E. Balsells , E. Wastnedge , et al., “Risk Factors for Respiratory Syncytial Virus Associated With Acute Lower Respiratory Infection in Children Under Five Years: Systematic Review and meta–Analysis,” Journal of Global Health 5 (2015): 020416. 10.7189/jogh.05.020416 PMC4676580 26682048

[5] C. B. Hall , G. A. Weinberg , M. K. Iwane , et al., “The Burden of Respiratory Syncytial Virus Infection in Young Children,” New England Journal of Medicine 360, no. 6 (2009): 588–598, 10.1056/nejmoa0804877. PMC4829966 19196675

[6] C. B. Hall , G. A. Weinberg , A. K. Blumkin , et al., “Respiratory Syncytial Virus–Associated Hospitalizations Among Children Less Than 24 Months of Age,” Pediatrics 132, no. 2 (2013): e341–e348, 10.1542/peds.2013-0303. 23878043

[7] B. Rha , A. T. Curns , J. Y. Lively , et al., “Respiratory Syncytial Virus–Associated Hospitalizations Among Young Children: 2015–2016,” Pediatrics 146, no. 1 (2020): e20193611, 10.1542/peds.2019-3611. PMC12874392 32546583

[8] C. S. Arriola , L. Kim , G. Langley , et al., “Estimated Burden of Community‐Onset Respiratory Syncytial Virus–Associated Hospitalizations Among Children Aged <2 Years in the United States, 2014–15,” Journal of the Pediatric Infectious Diseases Society 9, no. 5 (2019): 587–595, 10.1093/jpids/piz087. PMC7107566 31868913

[9] J. Henderson , T. N. Hilliard , A. Sherriff , et al., “Hospitalization for RSV Bronchiolitis Before 12 Months of Age and Subsequent Asthma, Atopy and Wheeze: A Longitudinal Birth Cohort Study,” Pediatric Allergy and Immunology 16 (2005): 386–392. 10.1111/j.1399-3038.2005.00298.x 16101930

[10] K. M. James , T. Gebretsadik , G. J. Escobar , et al., “Risk of Childhood Asthma Following Infant Bronchiolitis During the Respiratory Syncytial Virus Season,” Journal of Allergy and Clinical Immunology 132 (2013): 227–229. 10.1016/j.jaci.2013.01.009 PMC3677032 23419541

[11] A. J. Driscoll , S. H. Arshad , L. Bont , et al., “Does Respiratory Syncytial Virus Lower Respiratory Illness in Early Life Cause Recurrent Wheeze of Early Childhood and Asthma? Critical Review of the Evidence and Guidance for Future Studies From a World Health Organization‐Sponsored Meeting,” Vaccine 38 (2020): 2435–2448. 10.1016/j.vaccine.2020.01.020 PMC7049900 31974017

[12] M. O. Blanken , M. M. Rovers , J. M. Molenaar , et al., “Respiratory Syncytial Virus and Recurrent Wheeze in Healthy Preterm Infants,” New England Journal of Medicine 368 (2013): 1791–1799. 10.1056/NEJMoa1211917 23656644

[13] N. M. Scheltema , E. E. Nibbelke , J. Pouw , et al., “Respiratory Syncytial Virus Prevention and Asthma in Healthy Preterm Infants: A Randomised Controlled Trial,” Lancet Respiratory Medicine 6 (2018): 257–264. 10.1016/S2213-2600(18)30055-9 29500030

[14] A. Haerskjold , L. Stokholm , M. Linder , et al., “Palivizumab Exposure and the Risk of Atopic Dermatitis, Asthma and Allergic Rhinoconjunctivitis: A Cross‐National, Population‐Based Cohort Study,” Pediatric Drugs 19 (2017): 155–164. 10.1007/s40272-017-0215-7 28188494

[15] K. N. Carroll , T. Gebretsadik , G. J. Escobar , et al., “Respiratory Syncytial Virus Immunoprophylaxis in High‐Risk Infants and Development of Childhood Asthma,” Journal of Allergy and Clinical Immunology 139 (2017): 66–71.e3. 10.1016/j.jaci.2016.01.055 PMC5074917 27212083

[16] S. M. Brunwasser , B. M. Snyder , A. J. Driscoll , et al., “Assessing the Strength of Evidence for a Causal Effect of Respiratory Syncytial Virus Lower Respiratory Tract Infections on Subsequent Wheezing Illness: A Systematic Review and Meta‐Analysis,” Lancet Respiratory Medicine 8 (2020): 795–806. 10.1016/S2213-2600(20)30109-0 PMC7464591 32763206

[17] L. G. Stensballe , J. Brunbjerg Simonsen , S. F. Thomsen , et al., “The Causal Direction in the Association Between Respiratory Syncytial Virus Hospitalization and Asthma,” Journal of Allergy and Clinical Immunology 123 (2009): 131–137.e1. 10.1016/j.jaci.2008.10.042 19130934

[18] S. F. Thomsen , S. Van Der Sluis , L. G. Stensballe , et al., “Exploring the Association Between Severe Respiratory Syncytial Virus Infection and Asthma: A Registry‐Based Twin Study,” American Journal of Respiratory and Critical Care Medicine 179 (2009): 1091–1097. 10.1164/rccm.200809-1471OC 19286626

[19] K. Zomer‐Kooijker , C. S. P. M. Uiterwaal , A. C. Van Der Gugten , B. Wilbrink , L. J. Bont , and C. K. Van Der Ent , “Decreased Lung Function Precedes Severe Respiratory Syncytial Virus Infection and Post‐Respiratory Syncytial Virus Wheeze in Term Infants,” European Respiratory Journal 44 (2014): 666–674. 10.1183/09031936.00009314 24993909

[20] X.‐B. Zhang , L.‐J. Liu , L.‐L. Qian , et al., “Clinical Characteristics and Risk Factors of Severe Respiratory Syncytial Virus‐Associated Acute Lower Respiratory Tract Infections in Hospitalized Infants,” World Journal of Pediatrics 10 (2014): 360–364. 10.1007/s12519-014-0513-x 25515808

[21] L. Palmer , C. B. Hall , J. P. Katkin , et al., “Respiratory Outcomes, Utilization and Costs 12 Months Following a Respiratory Syncytial Virus Diagnosis Among Commercially Insured Late‐Preterm Infants,” Current Medical Research and Opinion 27 (2011): 403–412. 10.1185/03007995.2010.542744 21192761

[22] D. Prais , E. Kaplan , G. Klinger , et al., “Short‐ and Long‐Term Pulmonary Outcome of Palivizumab in Children Born Extremely Prematurely,” Chest 149 (2016): 801–808. 10.1378/chest.15-0328 26226546

[23] H. Mochizuki , S. Kusuda , K. Okada , et al., “Palivizumab Prophylaxis in Preterm Infants and Subsequent Recurrent Wheezing. Six‐Year Follow‐Up Study,” American Journal of Respiratory and Critical Care Medicine 196 (2017): 29–38. 10.1164/rccm.201609-1812OC 28152315

[24] S. Yoshihara , S. Kusuda , H. Mochizuki , K. Okada , S. Nishima , and S. EAF , “Effect of Palivizumab Prophylaxis on Subsequent Recurrent Wheezing in Preterm Infants,” Pediatrics 132 (2013): 811–818. 10.1542/peds.2013-0982 24127479

[25] K. N. Carroll , P. Wu , T. Gebretsadik , et al., “The Severity‐Dependent Relationship of Infant Bronchiolitis on the Risk and Morbidity of Early Childhood Asthma,” Journal of Allergy and Clinical Immunology 123 (2009): 1055–1061.e1. 10.1016/j.jaci.2009.02.021 PMC2703291 19361850

[26] L. I. Tapia , S. Ampuero , M. A. Palomino , et al., “Respiratory Syncytial Virus Infection and Recurrent Wheezing in Chilean Infants: A Genetic Background?,” Infection, Genetics and Evolution 16 (2013): 54–61. 10.1016/j.meegid.2012.12.030 23333336

[27] M. Eriksson , R. Bennet , and A. Nilsson , “Wheezing Following Lower Respiratory Tract Infections With Respiratory Syncytial Virus and Influenza A in Infancy,” Pediatric Allergy and Immunology 11 (2000): 193–197. 10.1034/j.1399-3038.2000.00076.x 10981530

[28] S. Bosis , S. Esposito , H. G. M. Niesters , et al., “Role of Respiratory Pathogens in Infants Hospitalized for a First Episode of Wheezing and Their Impact on Recurrences,” Clinical Microbiology and Infection 14 (2008): 677–684. 10.1111/j.1469-0691.2008.02016.x PMC7130007 18558940

[29] C. Rosas‐Salazar , T. Chirkova , T. Gebretsadik , et al., “Respiratory Syncytial Virus Infection During Infancy and Asthma During Childhood in the USA (INSPIRE): A Population‐Based, Prospective Birth Cohort Study,” Lancet 401 (2023): 1669–1680. 10.1016/S0140-6736(23)00811-5 PMC10367596 37086744

[30] P. Wu , W. D. Dupont , M. R. Griffin , et al., “Evidence of a Causal Role of Winter Virus Infection During Infancy in Early Childhood Asthma,” American Journal of Respiratory and Critical Care Medicine 178 (2008): 1123–1129. 10.1164/rccm.200804-579OC PMC2588491 18776151

[31] C. Almqvist , S. Ekberg , S. Rhedin , F. Fang , T. Fall , and C. Lundholm , “Season of Birth, Childhood Asthma and Allergy in a Nationwide Cohort—Mediation Through Lower Respiratory Infections,” Clinical & Experimental Allergy 50 (2020): 222–230. 10.1111/cea.13542 31782836

[32] W.‐C. Chang , K. D. Yang , M. Wu , et al., “Close Correlation Between Season of Birth and the Prevalence of Bronchial Asthma in a Taiwanese Population,” PLoS One 8 (2013): e80285. 10.1371/journal.pone.0080285 PMC3835889 24278271

[33] S. A. Buchan , H. Chung , N. Daneman , et al., “Estimating the Incidence of First RSV Hospitalization in Children Born in Ontario, Canada,” Journal of the Pediatric Infectious Diseases Society 12 (2023): 421–430. 10.1093/jpids/piad045 PMC10389057 37335754

[34] Statistics Canada , “Table 17‐10‐0005‐01 Population Estimates on July 1, by Age and Gender,” accessed March 2026, 10.25318/1710000501-eng.

[35] Statistics Canada , “Table 13‐10‐0429‐01 Live Births and Fetal Deaths (Stillbirths), by Place of Birth (Hospital or Non‐Hospital),” accessed March 2026, 10.25318/1310042901-eng.

[36] ICES , “ICES Data Dictionary,” accessed July 2026, https://datadictionary.ices.on.ca/Applications/DataDictionary/Default.aspx.

[37] S. A. Swanson and M. A. Hernán , “Commentary: How to Report Instrumental Variable Analyses (Suggestions Welcome),” Epidemiology 24 (2013): 370–374. 10.1097/EDE.0b013e31828d0590 23549180

[38] M. A. Hernán and J. M. Robins , “Instruments for Causal Inference: An Epidemiologist's Dream?,” Epidemiology 17 (2006): 360–372. 10.1097/01.ede.0000222409.00878.37 16755261

[39] A. Pisesky , E. I. Benchimol , C. A. Wong , et al., “Incidence of Hospitalization for Respiratory Syncytial Virus Infection Amongst Children in Ontario, Canada: A Population‐Based Study Using Validated Health Administrative Data,” PLoS One 11 (2016): e0150416. 10.1371/journal.pone.0150416 PMC4784925 26958849

[40] M. Chadha , S. Hirve , C. Bancej , et al., “Human Respiratory Syncytial Virus and Influenza Seasonality Patterns—Early Findings From the WHO Global Respiratory Syncytial Virus Surveillance,” Influenza and Other Respiratory Viruses 14, no. 6 (2020): 638–646, 10.1111/irv.12726. PMC7578323 32163226

[41] H. Nair , W. A. Brooks , M. Katz , et al., “Global Burden of Respiratory Infections due to Seasonal Influenza in Young Children: A Systematic Review and Meta‐Analysis,” Lancet 378 (2011): 1917–1930. 10.1016/S0140-6736(11)61051-9 22078723

[42] M. A. Hamilton , A. Calzavara , S. D. Emerson , et al., “Validating International Classification of Disease 10th Revision Algorithms for Identifying Influenza and Respiratory Syncytial Virus Hospitalizations,” PLoS One 16 (2021): e0244746. 10.1371/journal.pone.0244746 PMC7790248 33411792

[43] M. Lipsitch , E. Tchetgen Tchetgen , and T. Cohen , “Negative Controls: A Tool for Detecting Confounding and Bias in Observational Studies,” Epidemiology 21 (2010): 383–388. 10.1097/EDE.0b013e3181d61eeb PMC3053408 20335814

[44] S. E. Wilson , S. L. Deeks , and L. C. Rosella , “Importance of ICD‐10 Coding Directive Change for Acute Gastroenteritis (Unspecified) for Rotavirus Vaccine Impact Studies: Illustration From a Population‐Based Cohort Study From Ontario, Canada,” BMC Research Notes 8 (2015): 439. 10.1186/s13104-015-1412-5 PMC4570676 26374397

[45] A. S. Gershon , C. Wang , J. Guan , J. Vasilevska‐Ristovska , L. Cicutto , and T. To , “Identifying Patients With Physician‐Diagnosed Asthma in Health Administrative Databases,” Canadian Respiratory Journal 16 (2009): 183–188. 10.1155/2009/963098 PMC2807792 20011725

[46] T. To , S. Dell , P. T. Dick , et al., “Case Verification of Children With Asthma in Ontario,” Pediatric Allergy and Immunology 17 (2006): 69–76. 10.1111/j.1399-3038.2005.00346.x 16426258

[47] A. S. Gershon , J. Guan , and C. Wang , “Trends in Asthma Prevalence and Incidence in Ontario, Canada, 1996‐2005: A Population Study,” American Journal of Epidemiology 172 (2010): 728–736. 10.1093/aje/kwq189 20716702

[48] J. A. Omand , J. L. Maguire , D. L. O'Connor , et al., “Agreement Between a Health Claims Algorithm and Parent‐Reported Asthma in Young Children,” Pediatric Pulmonology 54 (2019): 1547–1556. 10.1002/ppul.24432 31332948

[49] M. T. Caballero , M. H. Jones , R. A. Karron , et al., “The Impact of Respiratory Syncytial Virus Disease Prevention on Pediatric Asthma,” Pediatric Infectious Disease Journal 35 (2016): 820–822. 10.1097/INF.0000000000001167 27351360

[50] L. Valeri and V. W. TJ , “Mediation Analysis Allowing for Exposure–Mediator Interactions and Causal Interpretation: Theoretical Assumptions and Implementation With SAS and SPSS Macros,” Psychological Methods 18 (2013): 137–150. 10.1037/a0031034 PMC3659198 23379553

[51] M. A. Campitelli , M. Inoue , A. J. Calzavara , J. C. Kwong , and A. Guttmann , “Low Rates of Influenza Immunization in Young Children Under Ontario's Universal Influenza Immunization Program,” Pediatrics 129 (2012): e1421–e1430. 10.1542/peds.2011-2441 22585770

[52] S. E. Wilson , H. Chung , K. L. Schwartz , et al., “Rotavirus Vaccine Coverage and Factors Associated With Uptake Using Linked Data: Ontario, Canada,” PLoS One 13 (2018): e0192809. 10.1371/journal.pone.0192809 PMC5812625 29444167

[53] C. Leal Rodríguez , S. A. Shah , M. A. Rasmussen , et al., “The Infant Gut Virome Is Associated With Preschool Asthma Risk Independently of Bacteria,” Nature Medicine 30 (2024): 138–148. 10.1038/s41591-023-02685-x 38102298

[54] K. Levy , A. E. Hubbard , and J. N. Eisenberg , “Seasonality of Rotavirus Disease in the Tropics: A Systematic Review and Meta‐Analysis,” International Journal of Epidemiology 38 (2009): 1487–1496. 10.1093/ije/dyn260 PMC2800782 19056806

[55] R. M. D'Souza , G. Hall , and N. G. Becker , “Climatic Factors Associated With Hospitalizations for Rotavirus Diarrhoea in Children Under 5 Years of Age,” Epidemiology and Infection 136 (2008): 56–64. 10.1017/S0950268807008229 PMC2870768 17352836

[56] R. A. Etzel , “How Environmental Exposures Influence the Development and Exacerbation of Asthma,” Pediatrics 112 (2003): 233–239. 12837915

