# Supplementary material for: Exploring the Relationship Between Respiratory Syncytial Virus and Asthma Using Calendar Time at Birth as a Source of Quasi‐Random Variation
Source: Paediatr Perinat Epidemiol. 2026 Aug 20;40(7):811–23. doi: 10.1111/ppe.70168 (PMC13624515; doi:10.1111/ppe.70168)
Supplement: Supplementary file 1 [file PPE-40-811-s001.pdf]

Supplemental Material

Table of Contents:

Appendix 1: page 2
Figure S1: page 3
Table S1: pages 4-5
Figure S2: page 6
Table S2: page 7
Appendix 2: page 8
Appendix 3: page 8
Appendix 4: page 8
Figure S3: page 9
Figure S4: page 9
Figure S5: page 10
Figure S6: page 11
Table S3: pages 12-15
Figure S7: page 16
Table S4: pages 17-20
Figure S8: page 21
Figure S9: page 22
Figure S10: page 23
Figure S11: page 24
Table S5: page 25
References: page 26

Appendix 1. Description of study datasets

Registered Persons Database (RPDB)
The RPDB includes basic demographic information about each person that had an Ontario health card from April 1991 onward and is updated every month. Data include date of birth/death, sex, and period of time for which the person was eligible for publicly funded health care through the Ontario Health Insurance Plan. Importantly, the RPDB contains an encrypted Ontario health card number, termed the ICES Key Number (IKN), the unique identifier by which all other databases held by ICES are deterministically linked.[1]

Discharge Abstract Database (DAD)
Assembled by the Canadian Institute for Health Information (CIHI) and transferred to ICES on a regular basis, the DAD includes information from all hospital separation abstracts submitted to CIHI by acute care hospitals in Ontario. Specifically, one primary and up to 24 secondary diagnoses as classified by the Canadian Implementation of the International Classification of Disease 9th (ICD-9-CA) and/or 10th Revisions (ICD-10-CA) and interventions classified by the Canadian Classification of Health Interventions (CCI), are included in this database.[1,2]

Ontario Health Insurance Plan (OHIP) Claims Database
The OHIP Claims Database includes billing information submitted by all providers in Ontario from July 1991 onward and is updated every month. Importantly, this database includes a fee code for diagnosis/reason for visit for all outpatient visits to physicians in Ontario.[1]

Ontario Asthma Dataset
Derived by ICES and updated annually, the Ontario Asthma Dataset is a special subset of the linked RPDB, DAD, and OHIP.[1,3,4] Researchers that assembled the Ontario Asthma Dataset identified asthma cases by using an algorithm of ≥1 asthma-related hospitalisation (prior to 2002-2003, ICD-9-CA code: 493; from 2002-2003 onward, ICD-10-CA codes: J45 and J46) and/or ≥2 asthma-related outpatient visits (ICD-9-CA code: 493) within two years.[3] Sensitivity and specificity of this algorithm to identify asthma cases in these data are 84% and 77%, respectively, compared to expert panel assessment of charts and among adults, 81% and 81%, respectively, compared to primary care physician diagnosis and among adults,[3] and 81% and 90%, respectively, compared to parent-reported asthma and among young children aged 1-5 years.[5]

MOMBABY
Derived by ICES, MOMBABY is a special subset of the DAD, which includes linked pregnant person-newborn delivery records for all hospital-based births that occurred from April 1988 onward in Ontario. Pregnant person and infant IKNs are available and linked for each delivery. Available information includes pregnant person/pregnancy characteristics (i.e., pregnant person age, number of previous live births, and plurality) and infant characteristics (i.e., sex, date of birth, gestational age at birth, and birthweight).[1] Gestational age is only available from 2002 onward, but an algorithm for estimating gestational age categories in prior years has recently been validated.[6]

Other Details
ICES is an independent, non-profit research institute whose legal status under Ontario's health information privacy law allows it to collect and analyze health care and demographic data, without consent, for health system evaluation and improvement. All data were linked (Figure S1) using unique encoded identifiers and analyzed within the secure network environment at ICES. Please visit https://www.ices.on.ca/ for further information.

Figure S1. Relationship/linkage between study datasets.

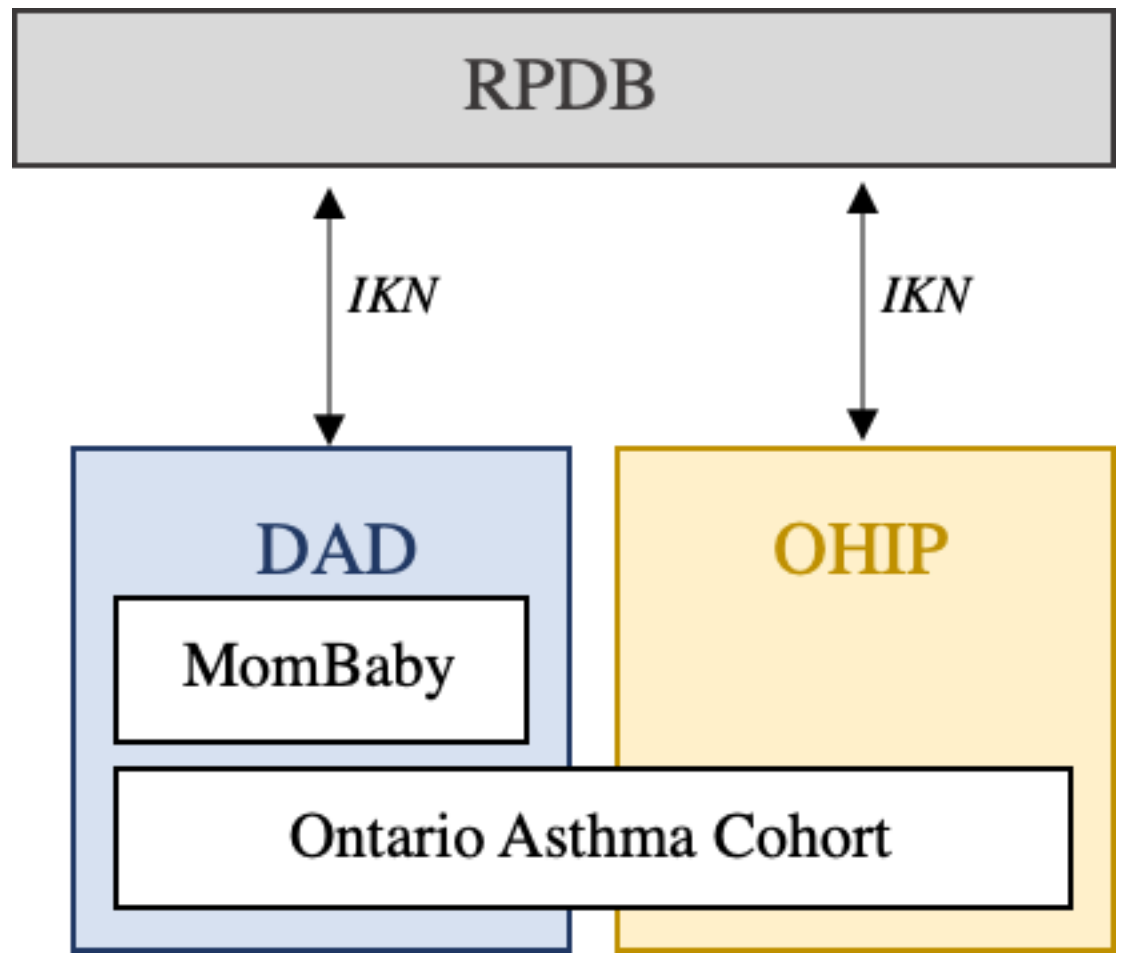

IKN (ICES Key Number) is a unique encrypted identifier that is used to link unique individuals across datasets.

Table S1. Summary of study datasets.

Dataset	Holder	Dates	Identifier(s)	Criteria	Key Variables	Original Source
Registered Persons Database (RPDB)	ICES	April 1991 onward	ICES key number (IKN)	• Includes anyone with Ontario Health Card	• Date of birth[a]<br>• Date of death[a]<br>• Eligibility time period<br>• Sex	N/A
Discharge Abstract Database (DAD)	CIHI (transferred to ICES annually)	April 1988 onward	ICES key number (IKN)	• Includes all hospital separation abstracts submitted by acute care hospitals in Ontario	• Medical diagnoses<br>○ Primary<br>○ Secondary (up to 24)<br>○ Canadian Implementation of the International Classification of Diseases, 9th Revision (ICD-9-CA)<br>○ Canadian Implementation of the International Classification of Diseases, 10th Revision (ICD-10-CA)<br>• Interventions<br>○ CCI (Canadian Classification of Health Interventions)	N/A
Ontario Health Insurance Plan (OHIP) Claims Database	ICES	July 1991 onward	ICES key number (IKN)	• Includes all claims submitted by providers and reimbursed by OHIP	• Medical diagnosis ("underlying reason for medical visit")	N/A
Ontario Asthma Dataset	ICES	April 1993/ 1996 onward	ICES key number (IKN)	• Includes all prevalent asthma cases from 1993 and incident asthma cases from 1996 identified by validated algorithm	• Source of diagnosis<br>• Incident diagnosis date	OHIP, DAD

Dataset	Holder	Dates	Identifier(s)	Criteria	Key Variables	Original Source
MOMBABY	CIHI (transferred to ICES annually)	April 1988 onward	Maternal/infant ICES key number (IKN)	• Includes linked mom/ newborn inpatient delivery records	• Infant date of birth<br>• Gestational age at delivery/birth<br>• Pregnant person age<br>• Birth weight<br>• Infant sex<br>• Number of previous live births<br>• Plurality	DAD

[a]*Identifiable variables used by ICES analysts to generate variables/datasets; removed prior to providing data to collaborators for analysis.*

Figure S2. Causal diagrams for the source of quasi-random variation presented throughout our manuscript (Panel A), as well as the potential roles of RSV infection in the absence of severe RSV LRTI (Panel B), seasonal factors (Panel C), and RSV vaccine (Panel D).

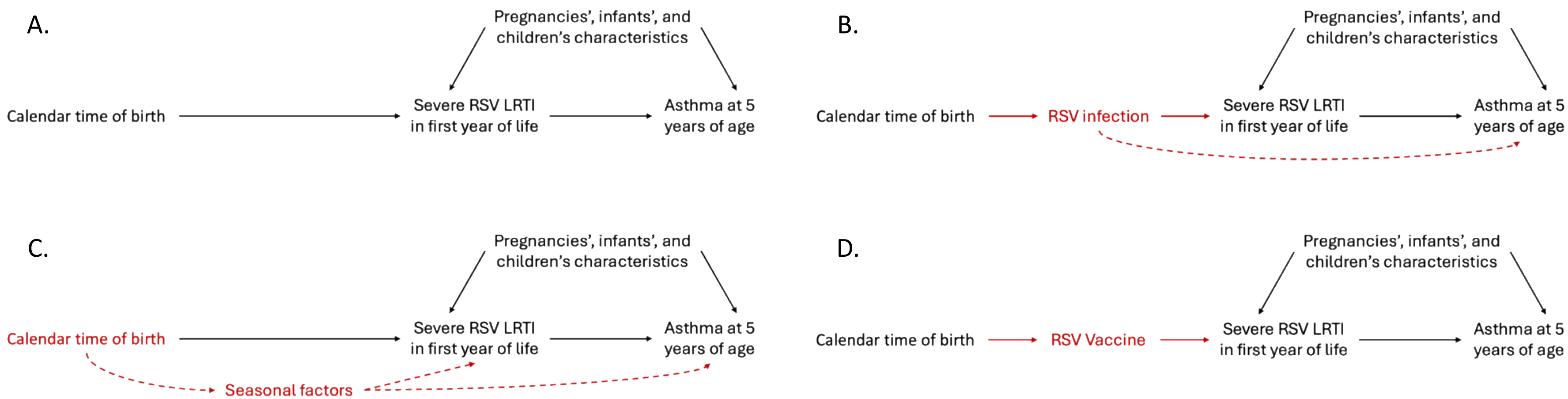

Table S2. Calendar weeks during which the highest number of RSV, influenza, and rotavirus hospitalisations occurred (i.e. definition of viral peaks).

Season [a]	RSV Peak Week	Influenza Peak Week	Rotavirus Peak Week
2002-2003	9 Feb 2003	5 Jan 2003	9 Mar 2003
2003-2004	29 Feb 2004	21 Dec 2003	21 Mar 2004
2004-2005	20 Feb 2005	13 Feb 2005	27 Mar 2005
2005-2006	19 Feb 2006	19 Mar 2006	26 Mar 2006
2006-2007	31 Dec 2006	28 Jan 2007	25 Mar 2007
2007-2008	03 Feb 2008	2 Mar 2008	24 Feb 2008
2008-2009	01 Feb 2009	8 Feb 2009	19 Apr 2009
2009-2010	31 Jan 2010	1 Nov 2009	4 Apr 2010
2010-2011	27 Feb 2011	16 Jan 2011	13 Feb 2011
2011-2012	01 Jan 2012	26 Feb 2012	18 Dec 2011
2012-2013	03 Mar 2013	23 Dec 2012	16 Dec 2012
2013-2014[b]	16 Feb 2014	5 Jan 2014	13 Jul 2014
2014-2015[b]	21 Dec 2014	21 Dec 2014	17 May 2015

[a]*Seasonal peak defined as week with highest number of virus-related hospitalisations among infants from September 1 to August 31 of the following year.*
[b]*Only relevant for sensitivity analyses in which the outcome definition did not require an inpatient/outpatient visit from 3-5 years of age among those diagnosed with asthma before 3 years of age for asthma.*

Appendix 2. Formulae for incidence of virus-related hospitalisations (i.e., proxy for seasonality) versus probability of virus-related hospitalisation.

Incidence of virus-related hospitalisation=
infants hospitalised with virus in a given calendar week /# infants aged <1 in a given calendar week

Probability of virus-related hospitalisation =
infants hospitalised with virus in the first year of life/# infants born in that calendar week

Appendix 3. Equations for regression analyses for objectives 1 and 2.

$$Logit(Y) = \sum \beta_{X_n} X_n + \sum \beta_{C_i} C_i$$

Equation 1. Y=asthma at 5 years of age (yes/no), X_n=vector for restricted cubic splines of infant age at RSV, influenza, or rotavirus peak (separately), C_i=vector for covariates. The unit of analysis was the infant.

$$Y = \sum \beta_{x_n} X_n$$

Equation 2. Y=probability of asthma at 5 years of age as a percentage of infants born in each calendar week, X_n=vector of incidences of RSV, influenza, and rotavirus hospitalisations among infants aged <1 year for each calendar week (offset by infant age at virus' peak that conferred the highest crude odds of paediatric asthma). The unit of analysis was the calendar week.

Appendix 4. Additional details about sensitivity analyses for Objective 1

For Objective 1, we calculated the probability of asthma at 5 years of age without requirement for an additional asthma-related visit among those diagnosed <3 years of age; calculated probability of asthma at 3 years of age; and calculated the probability of asthma at 6 years and at 8 years of age (among infants born up to March 2012 or March 2010, respectively). We adjusted for gestational age at birth and stratified models by term/preterm status, since gestational age at birth is a risk factor for both RSV infection and asthma yet might be on the causal pathway between calendar timing of birth and asthma. We included infants who died within the first 5 years of life in sensitivity analyses first by using asthma status recorded before infant death and second by classifying all such infants as having the outcome. We additionally specified knots at specific infant ages at RSV peak chosen from observed data (i.e., instead of placing knots at quantiles of data) and stratified models by infant sex. We used unstructured covariance for clustering of infants by mother, except in situations with model convergence issues, for which we used exchangeable covariance.

Figure S3. Unadjusted predicted odds ratio of asthma at 5 years of age by infant age at first RSV (panel A), influenza (panel B), and rotavirus (panel C) peaks. Infant ages from 0 to 26 weeks are displayed and infants with <5 years of follow-up were excluded.

A.

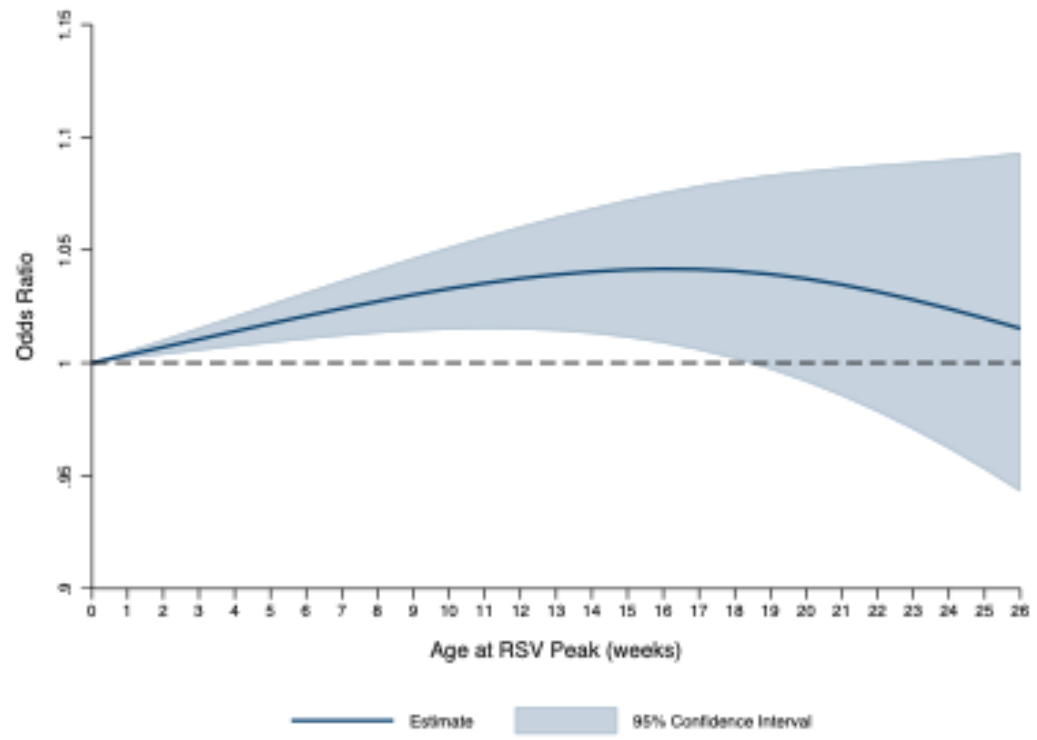

B.

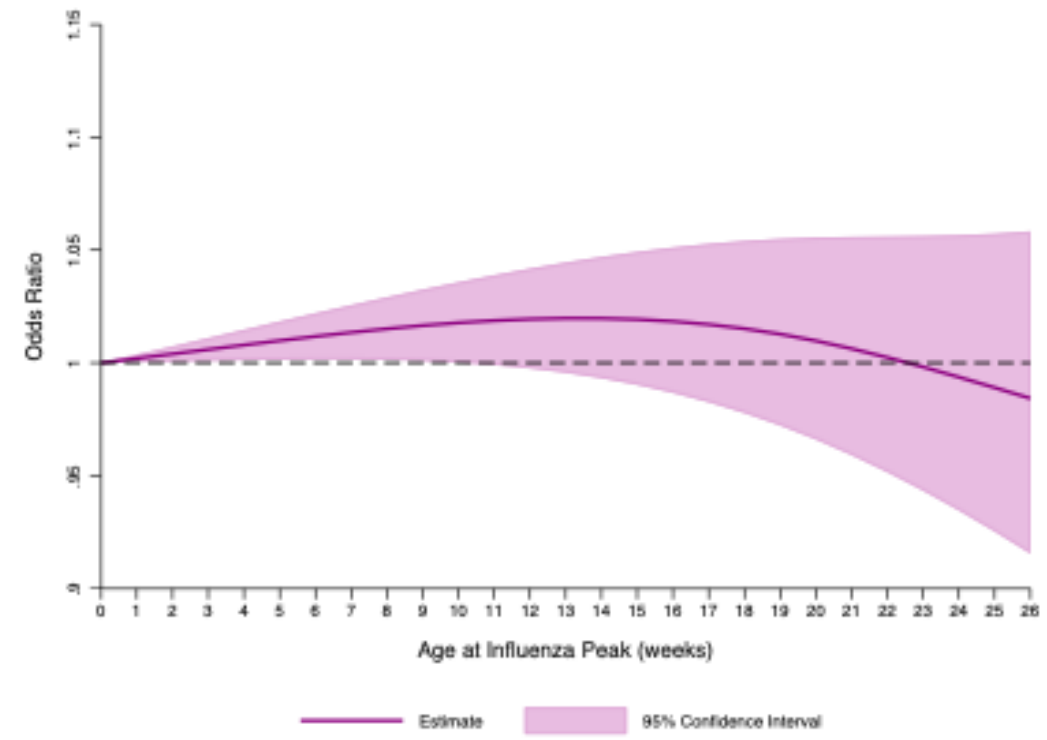

C.

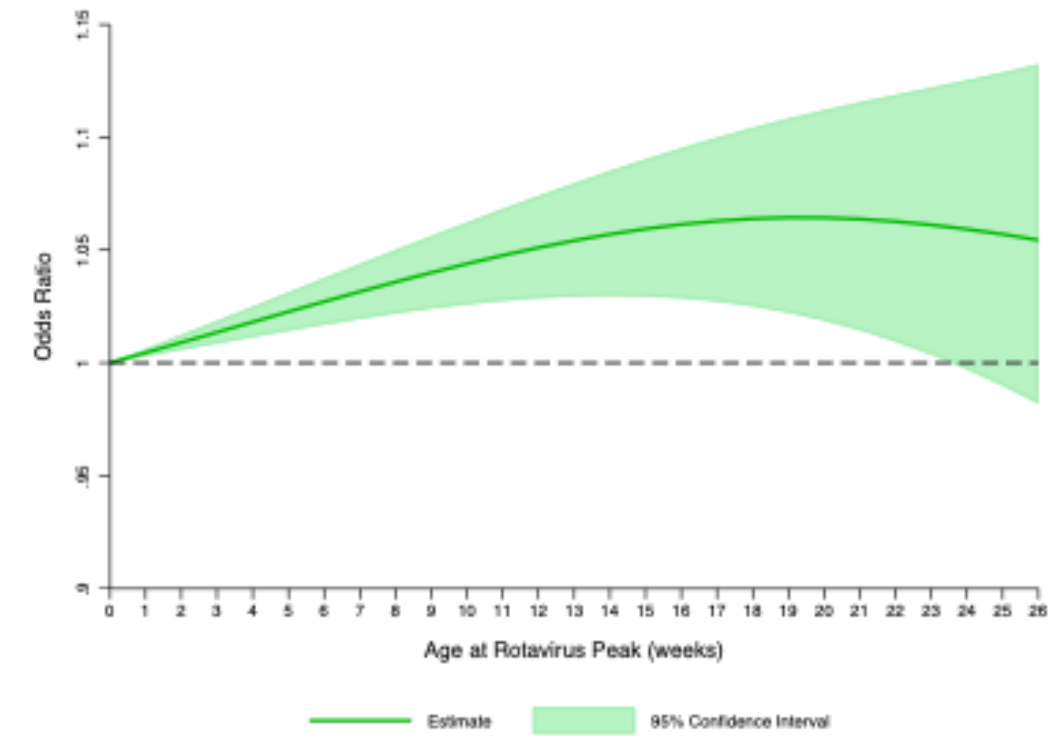

Figure S4. Unadjusted predicted odds ratio of asthma at 5 years of age by infant age at first RSV (panel A), influenza (panel B), and rotavirus (panel C) peaks. Infant ages from 0 to 52 weeks are displayed and infants with <5 years of follow-up were excluded.

A.

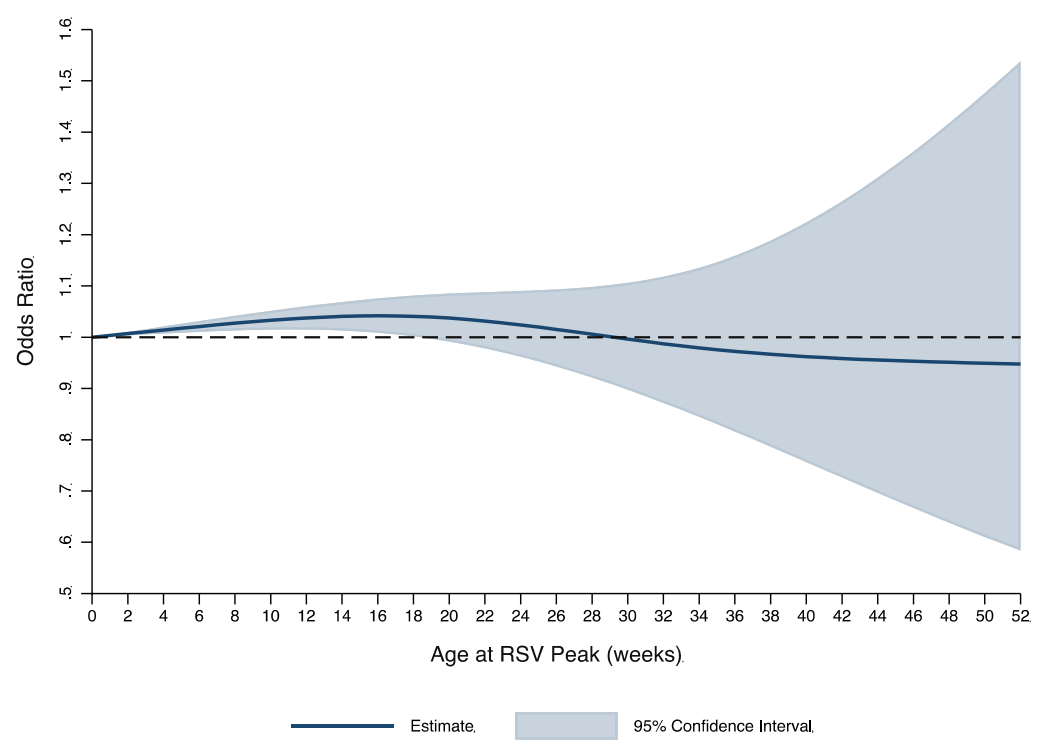

B.

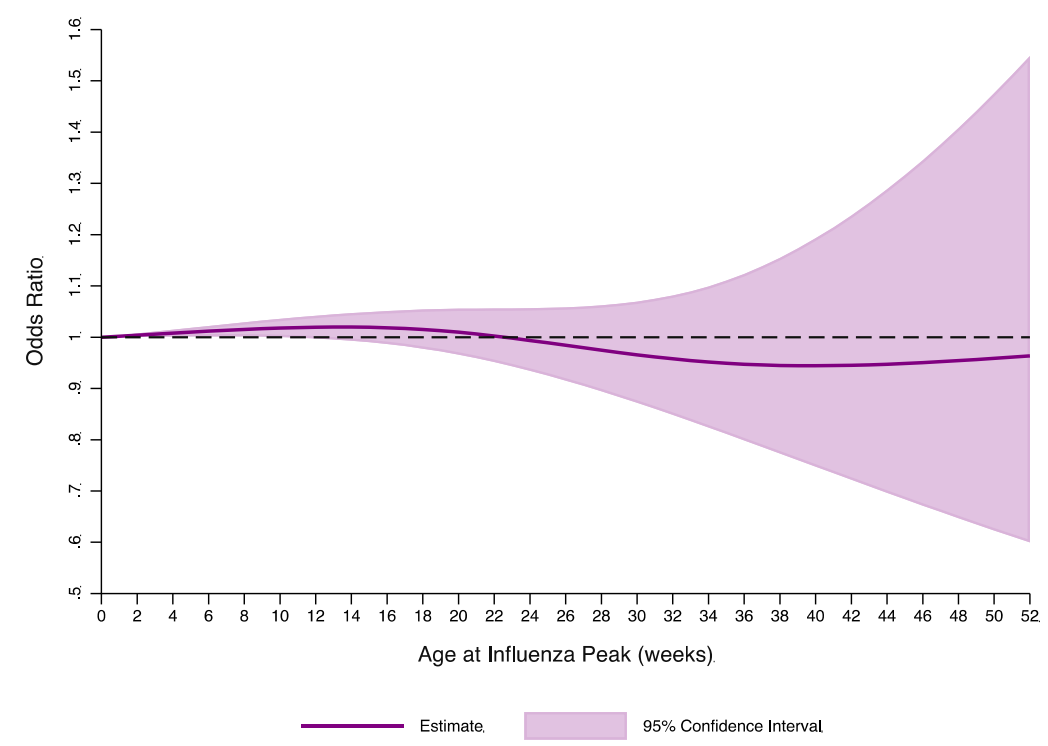

C.

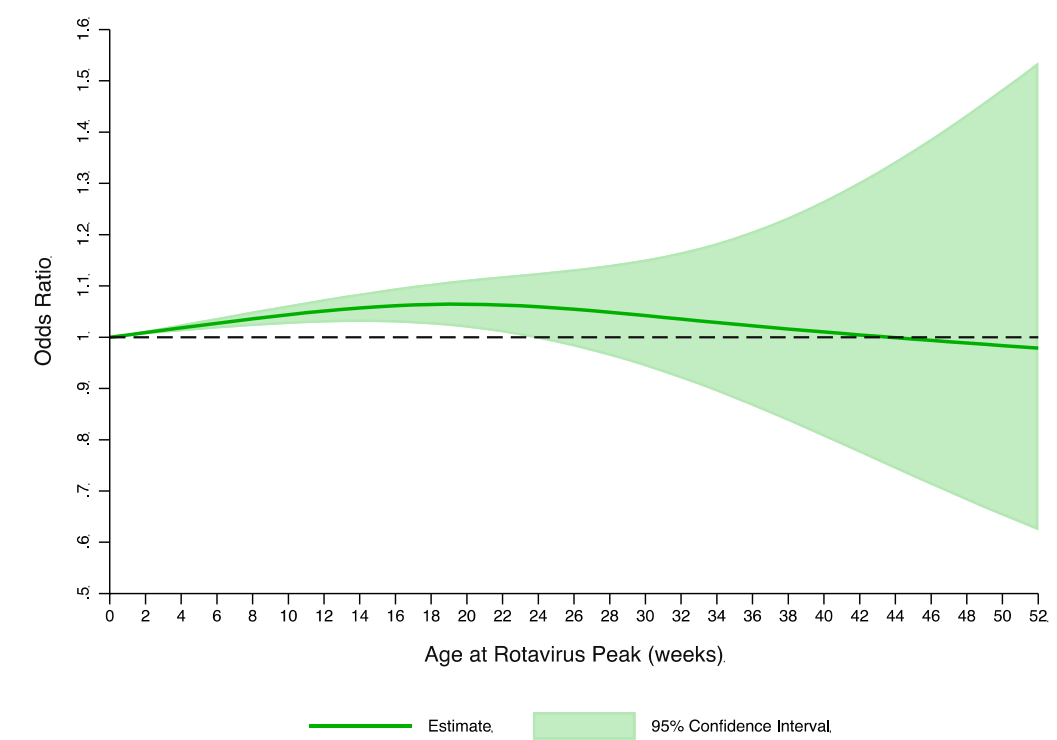

Figure S5. Adjusted predicted odds ratio of asthma at 5 years of age by infant age at first RSV (panel A), influenza (panel B), and rotavirus (panel C) peaks. Predicted odds ratios are adjusted for infant sex, pregnant person age, pregnant person asthma, previous live births, and virus season of birth. Infant ages from 0 to 52 weeks are displayed and infants with <5 years of follow-up were excluded.

A. B. C.

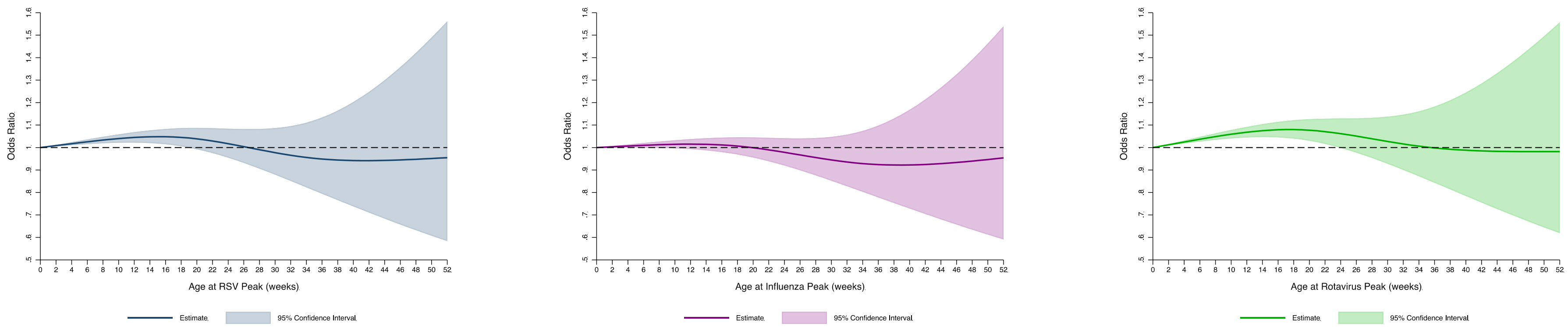

Figure S6. Predicted odds ratio of asthma at 5 years of age by pregnant person age modelled using splines (referent=youngest age). Infants with <5 years of follow-up were excluded.

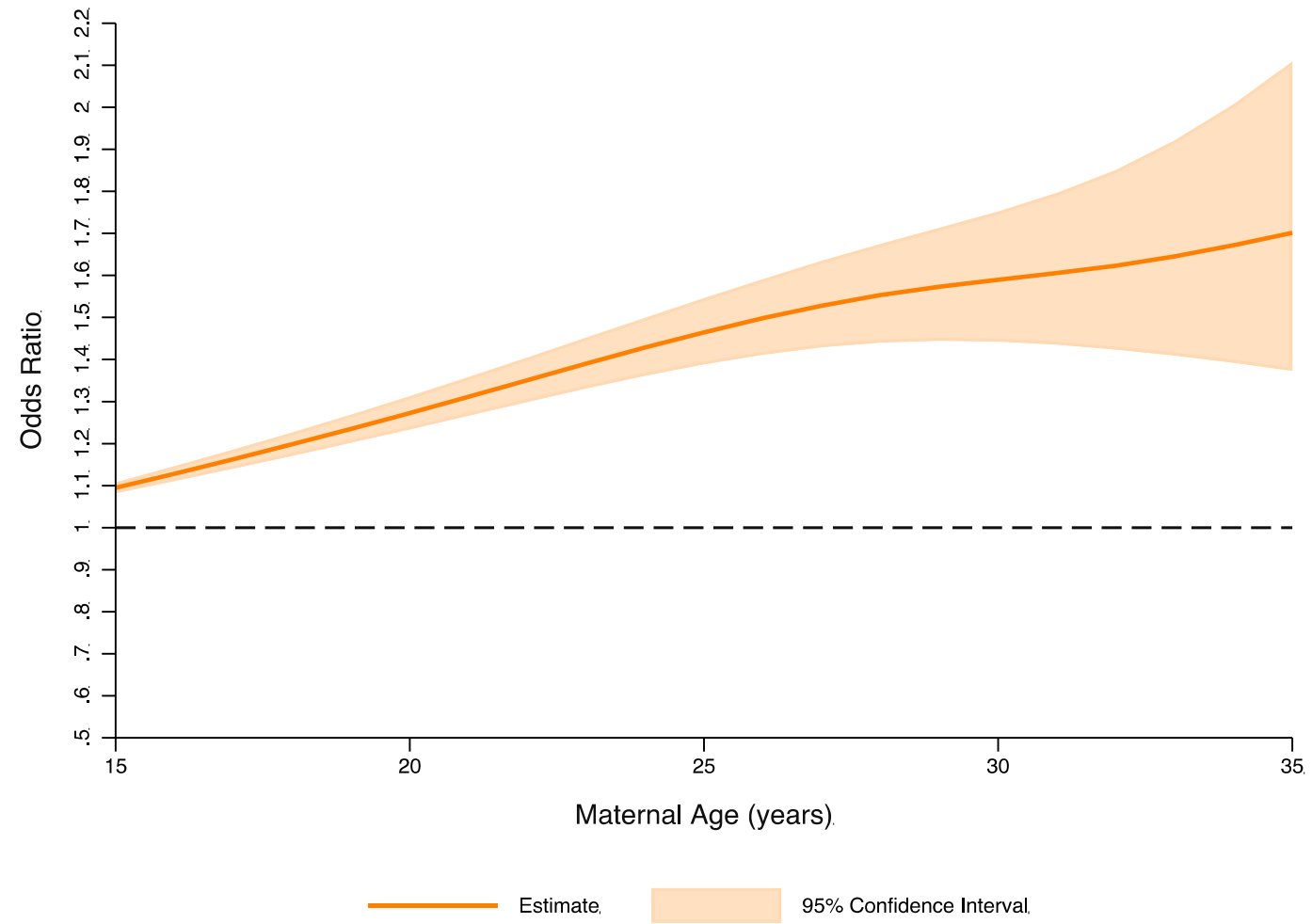

Table S3. Results from individual-level sensitivity analyses where the dependent variable was asthma at 5 years of age.

Model #	Model	N Infants	N Mothers	Predicted odds ratio of asthma at 5 years of age by infant age at RSV Peak (Infant Ages from 0-26 Weeks Displayed)	Coefficient for Infant Sex (Referent= Male)	Coefficient for Plurality Multiple (Referent= Singleton)	Coefficient for 1 previous live birth (Referent= 0)	Coefficient for >1 previous live birth (Referent= 0)	Coefficient for pregnant person asthma (Referent= No)	Coefficient for RSV year
1	Univariable, knots placed at quantiles (primary univariate)	1405595	925176	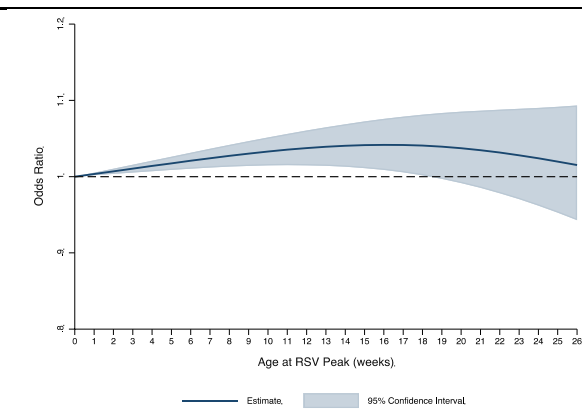	N/A	N/A	N/A	N/A	N/A	N/A
2	Multivariable, knots placed at quantiles (primary multivariable)[a]	1405595	925176	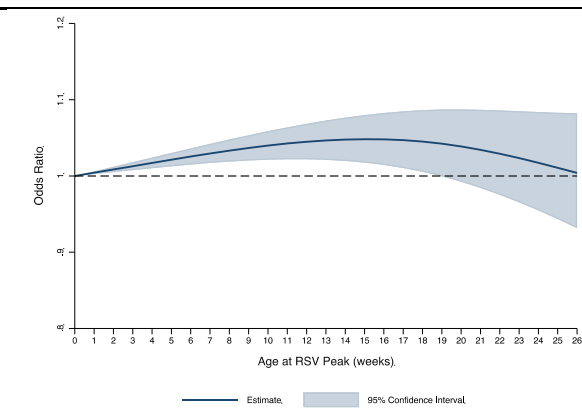	0.424 (0.413; 0.434)	0.121 (0.088; 0.154)	-0.134 (-0.146; -0.123)	-0.332 (-0.348; -0.316)	0.542 (0.527; 0.557)	-0.025 (-0.027; -0.024)
3	Knots placed at 13- and 31-weeks' infant age at RSV peak	1405595	925176	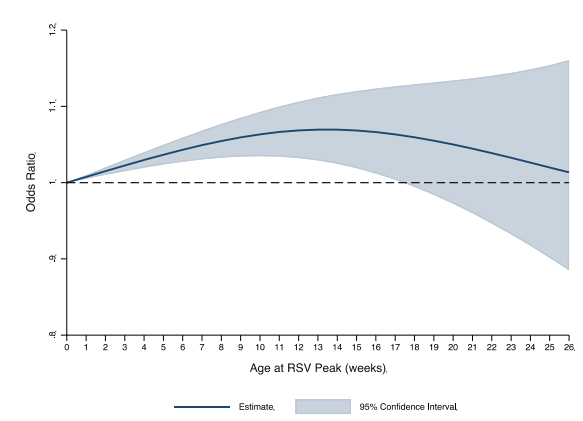	0.424 (0.413; 0.434)	0.121 (0.088; 0.154)	-0.134 (-0.146; -0.123)	-0.331 (-0.347; -0.315)	0.542 (0.527; 0.557)	-0.025 (-0.027; -0.024)
4	Includes infants with <5 years of follow-up	1437731	944822	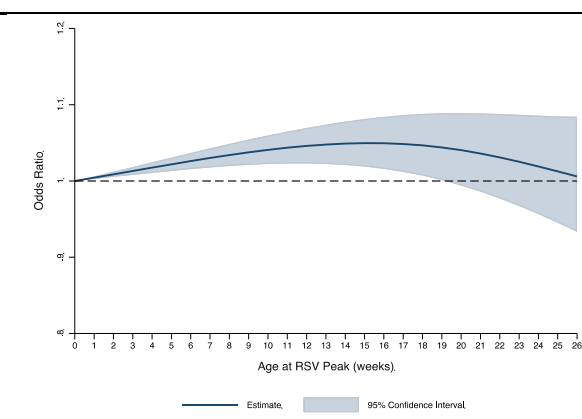	0.422 (0.411; 0.433)	0.097 (0.064; 0.129)	-0.133 (-0.144; -0.121)	-0.335 (-0.351; -0.319)	0.547 (0.532; 0.562)	-0.024 (-0.026; -0.023)

Model #	Model	N Infants	N Mothers	Predicted odds ratio of asthma at 5 years of age by infant age at RSV Peak (Infant Ages from 0-26 Weeks Displayed)	Coefficient for Infant Sex (Referent= Male)	Coefficient for Plurality Multiple (Referent= Singleton)	Coefficient for 1 previous live birth (Referent= 0)	Coefficient for >1 previous live birth (Referent= 0)	Coefficient for pregnant person asthma (Referent= No)	Coefficient for RSV year
5	Includes deaths	1411752	926809	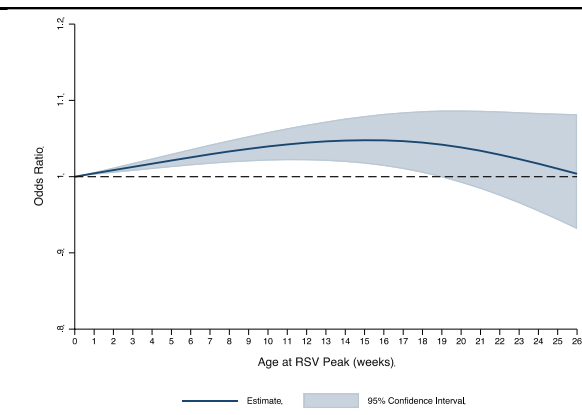	0.423 (0.412; 0.434)	0.096 (0.064; 0.129)	-0.133 (-0.145; -0.122)	-0.332 (-0.348; -0.316)	0.542 (0.527; 0.557)	-0.025 (-0.027; -0.023)
6	Includes deaths and classifies as asthma	1411752	926809	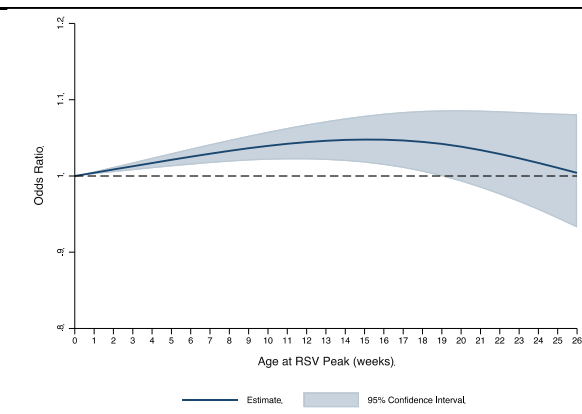	0.415 (0.405; 0.426)	0.251 (0.220; 0.282)	-0.138 (-0.150; -0.127)	-0.326 (-0.342; -0.310)	0.527 (0.512; 0.542)	-0.025 (-0.027; -0.024)
7	Excludes if infant age at RSV peak >52 weeks	1356859	904013	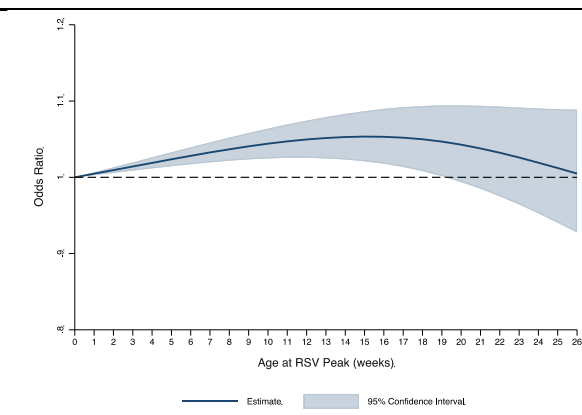	0.423 (0.413; 0.434)	0.121 (0.088; 0.154)	-0.136 (-0.147; -0.124)	-0.334 (-0.350; -0.318)	0.542 (0.527; 0.557)	-0.025 (-0.027; -0.023)
8	Uses secondary outcome definition (i.e. asthma at 5 years without 3-5 year criteria)[b]	1405595	925176	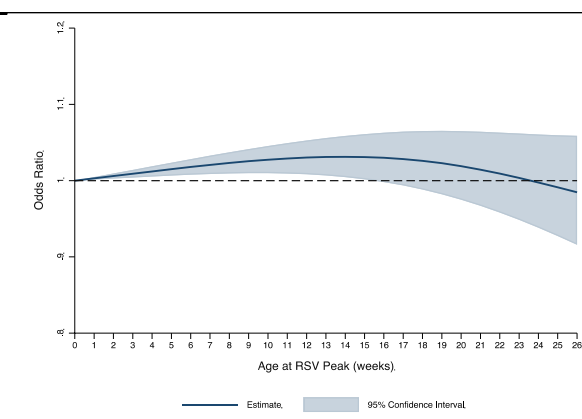	0.501 (0.490; 0.511)	0.199 (0.167; 0.231)	0.152 (0.141; 0.164)	0.111 (0.096; 0.126)	0.550 (0.536; 0.565)	-0.042 (-0.043; -0.040)
9	Uses secondary outcome definition (i.e. asthma by 3 years)[b]	1413902	930773	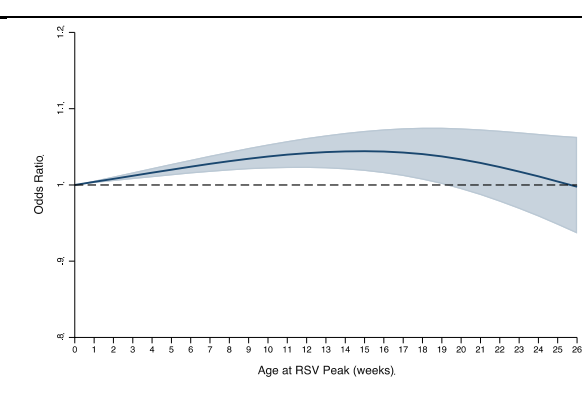	0.454 (0.445; 0.464)	0.169 (0.141; 0.197)	0.016 (0.006; 0.026)	-0.074 (-0.088; -0.061)	0.540 (0.527; 0.553)	-0.035 (-0.037; -0.034)

Model #	Model	N Infants	N Mothers	Predicted odds ratio of asthma at 5 years of age by infant age at RSV Peak (Infant Ages from 0-26 Weeks Displayed)	Coefficient for Infant Sex (Referent= Male)	Coefficient for Plurality Multiple (Referent= Singleton)	Coefficient for 1 previous live birth (Referent= 0)	Coefficient for >1 previous live birth (Referent= 0)	Coefficient for pregnant person asthma (Referent= No)	Coefficient for RSV year
10	Uses secondary outcome definition (i.e. asthma by 6 years)	1272908	855881	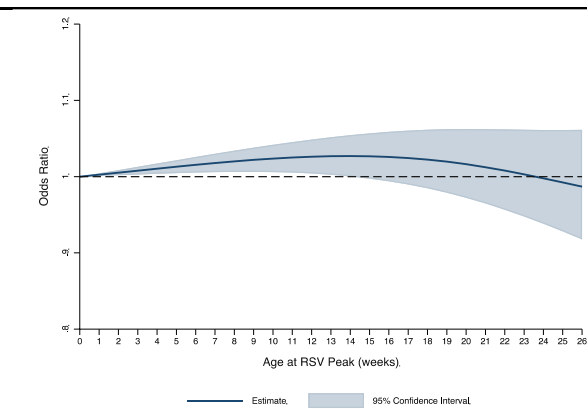	0.421 (0.411; 0.432)	0.102 (0.070; 0.134)	-0.142 (-0.153; -0.131)	-0.334 (-0.349; -0.318)	0.544 (0.530; 0.560)	-0.026 (-0.028; -0.024)
11	Uses secondary outcome definition (i.e. asthma by 8 years)	1007953	716245	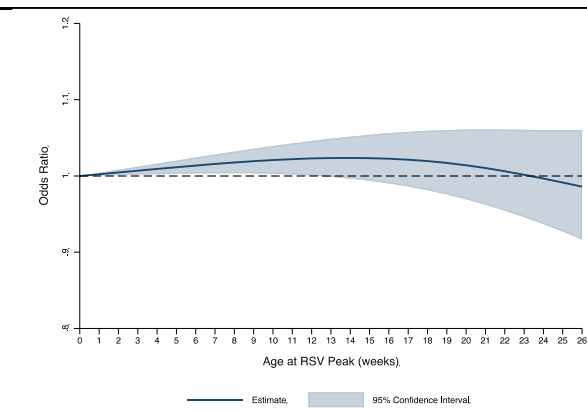	0.426 (0.415; 0.437)	0.098 (0.064; 0.132)	-0.145 (-0.156; -0.133)	-0.335 (-0.351; -0.318)	0.556 (0.540; 0.572)	-0.032 (-0.034; -0.029)
12	Controls for gestational age at birth[c]	1405385	925087	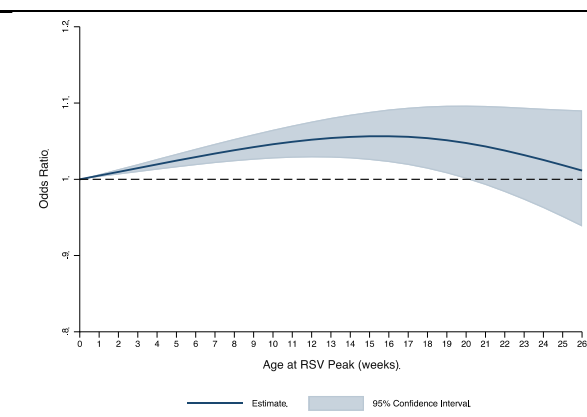	0.418 (0.407; 0.429)	-0.198 (-0.232; -0.163)	-0.138 (-0.150; -0.127)	-0.340 (-0.356; -0.323)	0.536 (0.521; 0.551)	-0.026 (-0.028; -0.025)
13	Restricted to preterm births[b]	108111	88310	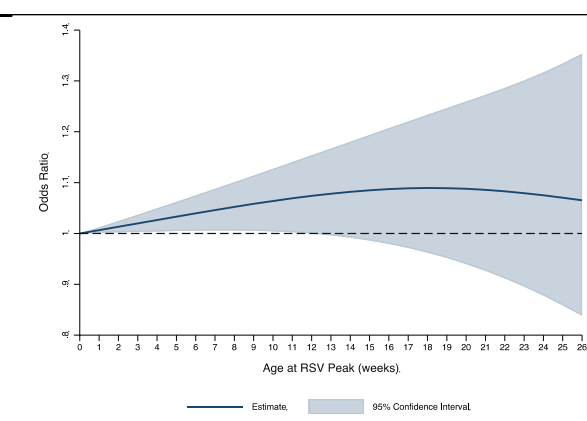	0.384 (0.349; 0.418)	-0.139 (-0.185; -0.092)	-0.147 (-0.186; -0.108)	-0.299 (-0.349; -0.249)	0.486 (0.441; 0.531)	-0.026 (-0.032; -0.020)
14	Restricted to term births	1194741	827854	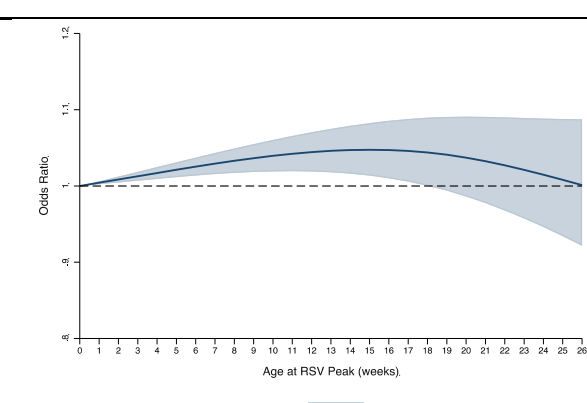	0.422 (0.410; 0.434)	-0.087 (-0.168; -0.006)	-0.127 (-0.140; -0.115)	-0.339 (-0.357; -0.321)	0.543 (0.527; 0.560)	-0.026 (-0.028; -0.024)

Model #	Model	N Infants	N Mothers	Predicted odds ratio of asthma at 5 years of age by infant age at RSV Peak (Infant Ages from 0-26 Weeks Displayed)	Coefficient for Infant Sex (Referent= Male)	Coefficient for Plurality Multiple (Referent= Singleton)	Coefficient for 1 previous live birth (Referent= 0)	Coefficient for >1 previous live birth (Referent= 0)	Coefficient for pregnant person asthma (Referent= No)	Coefficient for RSV year
15	Restricted to female infants	685261	558264	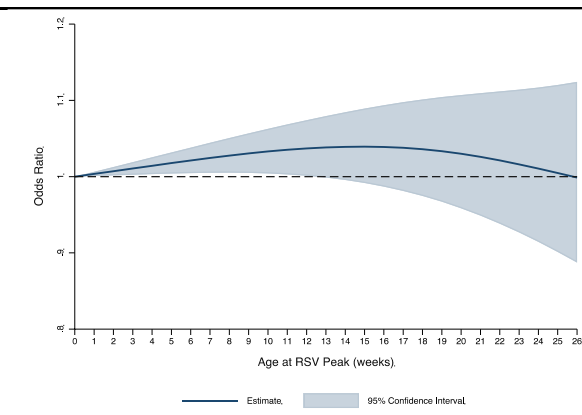	N/A	0.117 (0.069; 0.165)	-0.150 (-0.169; -0.132)	-0.381 (-0.407; -0.356)	0.576 (0.554; 0.599)	-0.022 (-0.024; -0.019)
16	Restricted to male infants	720279	582039	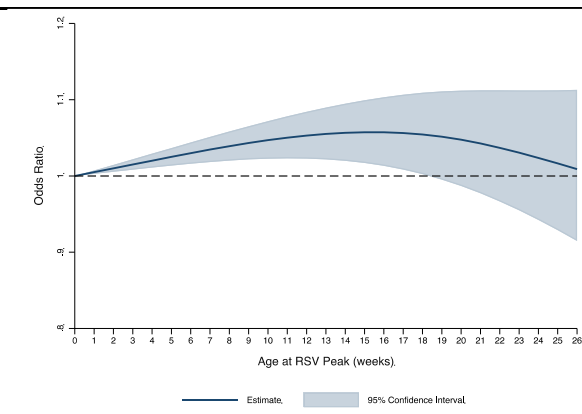	N/A	0.121 (0.080; 0.162)	-0.127 (-0.142; -0.111)	-0.317 (-0.338; -0.297)	0.523 (0.504; 0.543)	-0.028 (-0.030; -0.026)
17	Controls for RSV hospitalisation (yes/no)[d]	1405595	925176	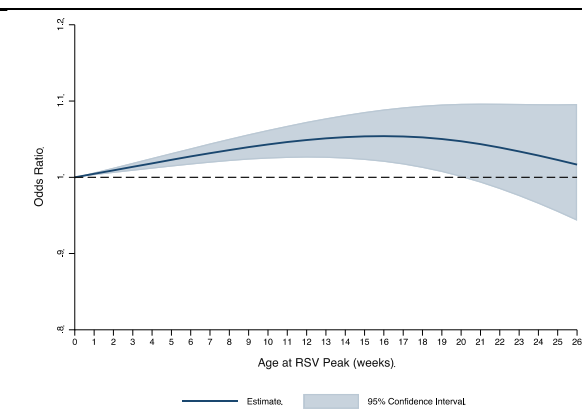	0.421 (0.410; 0.432)	0.110 (0.077; 0.143)	-0.145 (-0.157; -0.134)	-0.350 (-0.366; -0.334)	0.539 (0.523; 0.554)	-0.025 (-0.027; -0.024)

[a] *See Figure S6 for predicted odds ratio of asthma at 5 years of age by pregnant person age modelled using splines.*
[b] *Fit with exchangeable instead of unstructured covariance due to model convergence issues.*
[c] *See Figure S7 for predicted odds ratio of asthma at 5 years of age by gestational age at birth modelled using splines.*
[d] *Coefficient for RSV hospitalisation (yes/no): 0.891 (0.849; 0.932).*

Figure S7. Predicted odds ratio of asthma at 5 years of age by gestational age at birth modelled using splines (referent=youngest age). Infants with <5 years of follow-up and/or missing gestational age at birth were excluded.

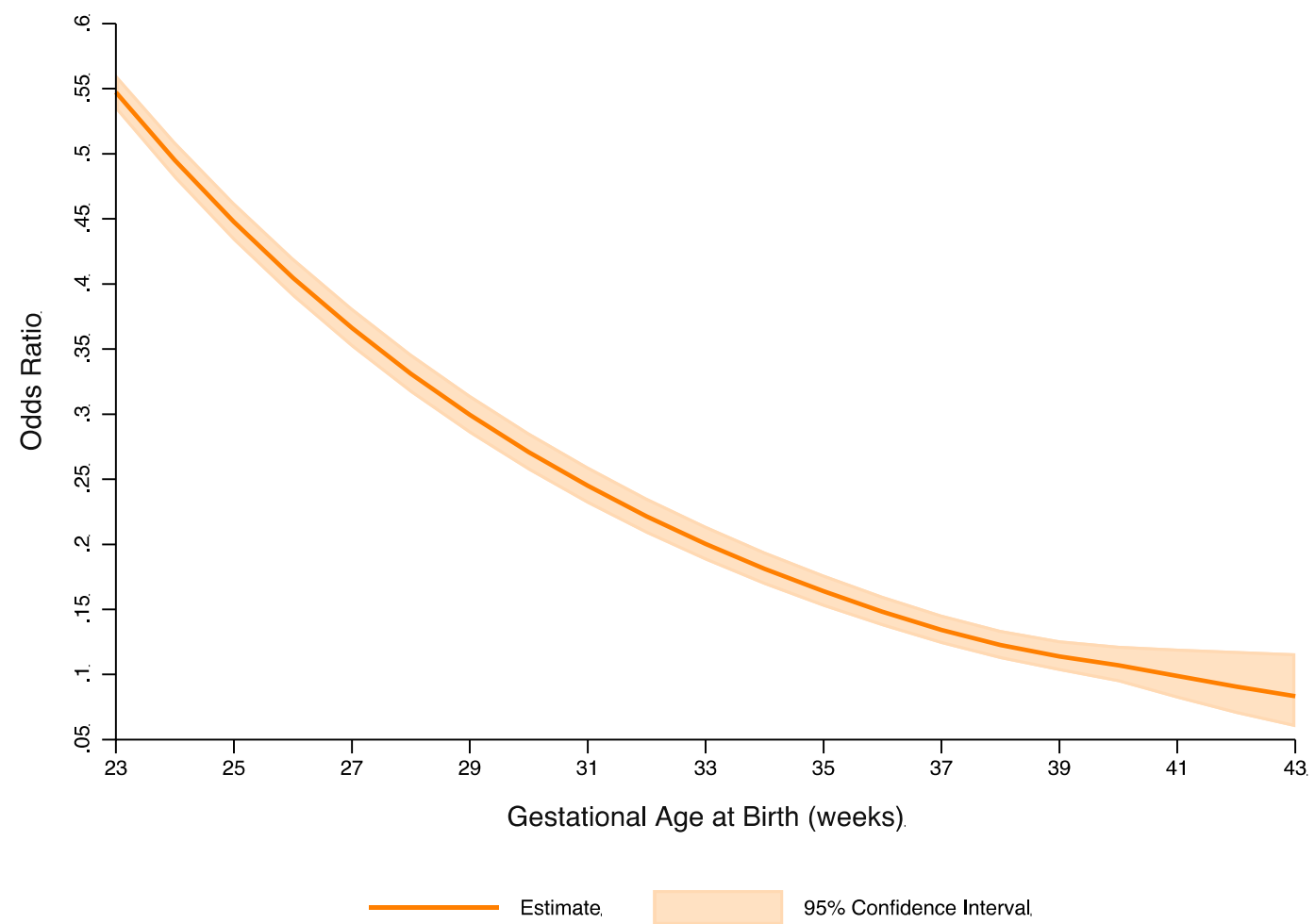

Table S4. Results from population-level sensitivity analyses where the dependent variable is probability of asthma at 5 years of age as a percentage of infants born in each calendar week.

Model #	Model	N Weeks	Coefficient for Constant	Coefficient for probability of RSV hospitalisation in infancy	Coefficient for probability of influenza hospitalisation in infancy	Coefficient for probability of rotavirus hospitalisation in infancy	Coefficient for weekly incidence of RSV hospitalisation	Coefficient for weekly incidence of influenza hospitalisation	Coefficient for weekly incidence of rotavirus hospitalisation
1	Probability of infection hospitalisation, without offset	552	0.094 (0.092; 0.097)	0.698 (0.566; 0.831)	-2.279 (-2.866; -1.692)	1.322 (1.002; 1.642)	N/A	N/A	N/A
2	Probability of infection hospitalisation, with offset	536	0.096 (0.093; 0.099)	0.225 (0.084; 0.366)	0.058 (-0.505; 0.620)	0.884 (0.569; 1.198)	N/A	N/A	N/A
3	Weekly incidence of infection hospitalisation, without offset	552	0.103 (0.101; 0.105)	N/A	N/A	N/A	-4.190 (-7.572; -0.809)	11.151 (-16.280; 38.582)	11.911 (1.965; 21.857)
4	Weekly incidence of infection hospitalisation with offset from crude odds	536	0.096 (0.094; 0.098)	N/A	N/A	N/A	3.149 (-0.068; 6.365)	19.710 (-4.037; 43.457)	45.502 (36.060; 54.944)
5	Weekly incidence of infection hospitalisation with offset from univariable model	533	0.097 (0.095; 0.099)	N/A	N/A	N/A	0.095 (-3.142; 3.331)	1.710 (-22.195; 25.615)	51.325 (41.820; 60.829)
6	Weekly incidence of infection hospitalisation with offset from multivariable model	535	0.097 (0.095; 0.098)	N/A	N/A	N/A	1.857 (-1.298; 5.013)	11.940 (-12.047; 35.927)	47.367 (38.105; 56.629)

Model #	Model	N Weeks	Coefficient for Constant	Coefficient for probability of RSV hospitalisation in infancy	Coefficient for probability of influenza hospitalisation in infancy	Coefficient for probability of rotavirus hospitalisation in infancy	Coefficient for weekly incidence of RSV hospitalisation	Coefficient for weekly incidence of influenza hospitalisation	Coefficient for weekly incidence of rotavirus hospitalisation
7	Weekly incidence of infection hospitalisation with offset from crude odds, linear year[a]	536	0.108 (0.105; 0.110)	N/A	N/A	N/A	6.394 (3.586; 9.203)	18.835 (-1.617; 39.286)	19.915 (10.989; 28.841)
8	Weekly incidence of infection hospitalisation with offset from crude odds, linear year, and binary preterm[b]	536	0.108 (0.100; 0.116)	N/A	N/A	N/A	6.385 (3.549; 9.221)	18.809 (-1.687; 39.305)	19.957 (10.872; 29.042)
9	Weekly incidence of RSV hospitalisation only without offset	552	0.105 (0.104; 0.106)	N/A	N/A	N/A	-2.325 (-5.355; 0.705)	N/A	N/A
10	Weekly incidence of RSV hospitalisation only with offset from crude odds	539	0.102 (0.101; 0.103)	N/A	N/A	N/A	12.009 (9.175; 14.843)	N/A	N/A
11	Weekly incidence of influenza hospitalisation only without offset	552	0.104 (0.102; 0.106)	N/A	N/A	N/A	N/A	7.988 (-19.500; 35.476)	N/A

Model #	Model	N Weeks	Coefficient for Constant	Coefficient for probability of RSV hospitalisation in infancy	Coefficient for probability of influenza hospitalisation in infancy	Coefficient for probability of rotavirus hospitalisation in infancy	Coefficient for weekly incidence of RSV hospitalisation	Coefficient for weekly incidence of influenza hospitalisation	Coefficient for weekly incidence of rotavirus hospitalisation
12	Weekly incidence of influenza hospitalisation only with offset from crude odds	541	0.103 (0.102; 0.105)	N/A	N/A	N/A	N/A	23.342 (-3.771; 50.456)	N/A
13	Weekly incidence of rotavirus hospitalisation only without offset	552	0.103 (0.102; 0.105)	N/A	N/A	N/A	N/A	N/A	6.292 (-2.629; 15.212)
14	Weekly incidence of rotavirus hospitalisation only with offset from crude odds	536	0.097 (0.096; 0.098)	N/A	N/A	N/A	N/A	N/A	50.889 (43.117; 58.661)
15	Probability of RSV hospitalisation in infancy and weekly incidence of RSV hospitalisation with offset from crude odds	539	0.100 (0.098; 0.101)	0.326 (0.160; 0.491)	N/A	N/A	7.809 (4.288; 11.329)	N/A	N/A

Model #	Model	N Weeks	Coefficient for Constant	Coefficient for probability of RSV hospitalisation in infancy	Coefficient for probability of influenza hospitalisation in infancy	Coefficient for probability of rotavirus hospitalisation in infancy	Coefficient for weekly incidence of RSV hospitalisation	Coefficient for weekly incidence of influenza hospitalisation	Coefficient for weekly incidence of rotavirus hospitalisation
16	Probability of influenza hospitalisation in infancy and weekly incidence of influenza hospitalisation with offset from crude odds	541	0.105 (0.103; 0.107)	N/A	-0.625 (-1.189; -0.061)	N/A	N/A	23.244 (-3.776; 50.264)	N/A
17	Probability of rotavirus hospitalisation in infancy and weekly incidence of rotavirus hospitalisation with offset from crude odds	536	0.094 (0.092; 0.096)	N/A	N/A	0.462 (0.181; 0.743)	N/A	N/A	50.041 (42.320; 57.763)

[a] *Coefficient for year =-0.002 (-0.002; -0.001)*

[b] *Coefficient for year = -0.002 (-0.002; -0.001), coefficient for preterm birth = -0.002 (-0.098; 0.093)*

Figure S8. Incidence of infection-related hospitalisations by calendar week and probability of preterm birth and asthma at 5 years of age by calendar week of birth. Incidence of infection-related hospitalisations by calendar week plotted without offset (Panel A) and with offset (Panel B) by age at which crude log-odds of asthma at age 5 was highest. The offset terms for Panel B were 13 weeks for RSV, 11 weeks for influenza, and 16 weeks for rotavirus. To facilitate visualisation in figures, incidence of influenza-related hospitalisations was set to a maximum of 12.5 per 10,000 infants if greater than this value (i.e. during the 2009-2010 A/H1N1 influenza pandemic).

A.

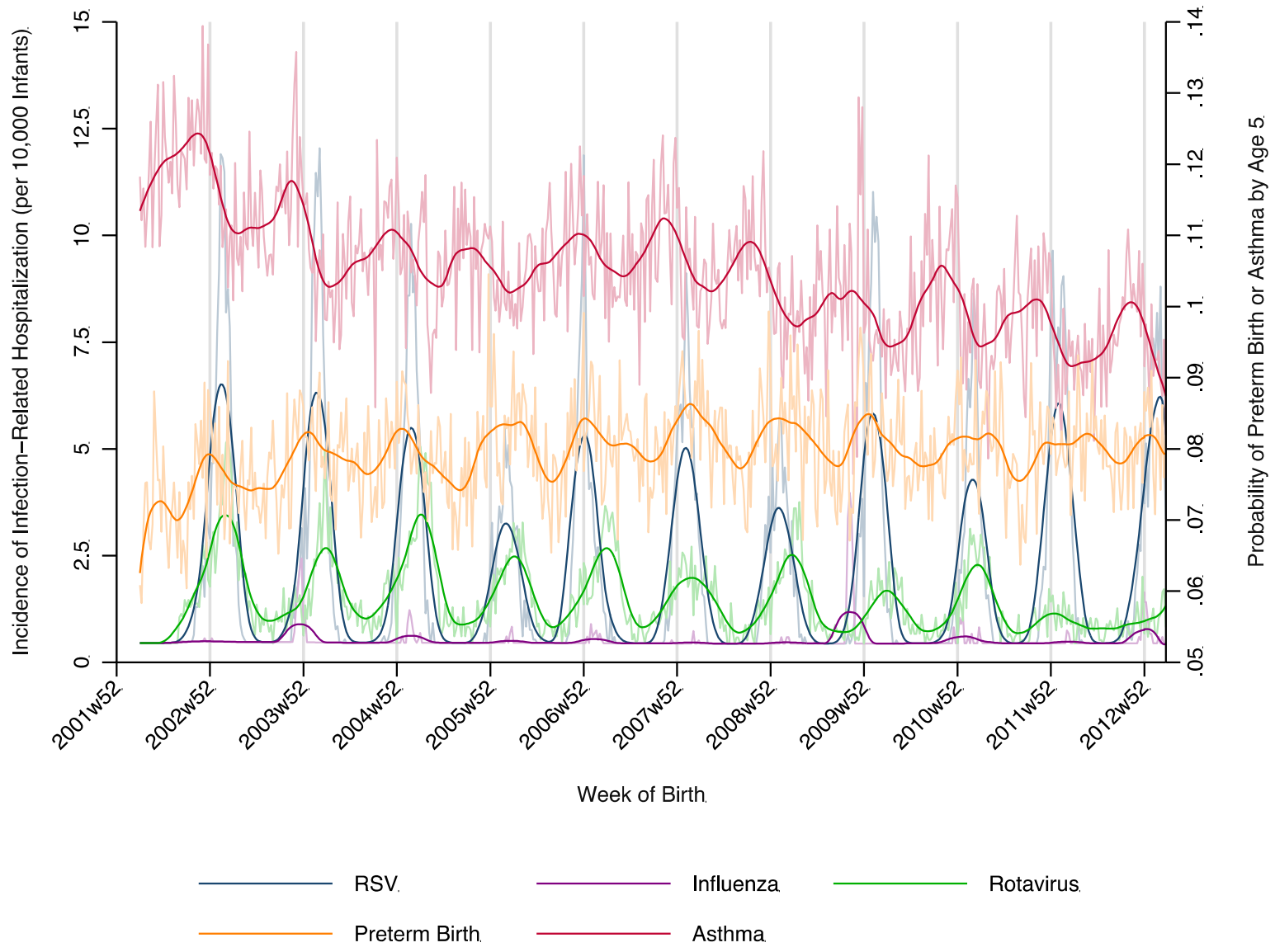

B.

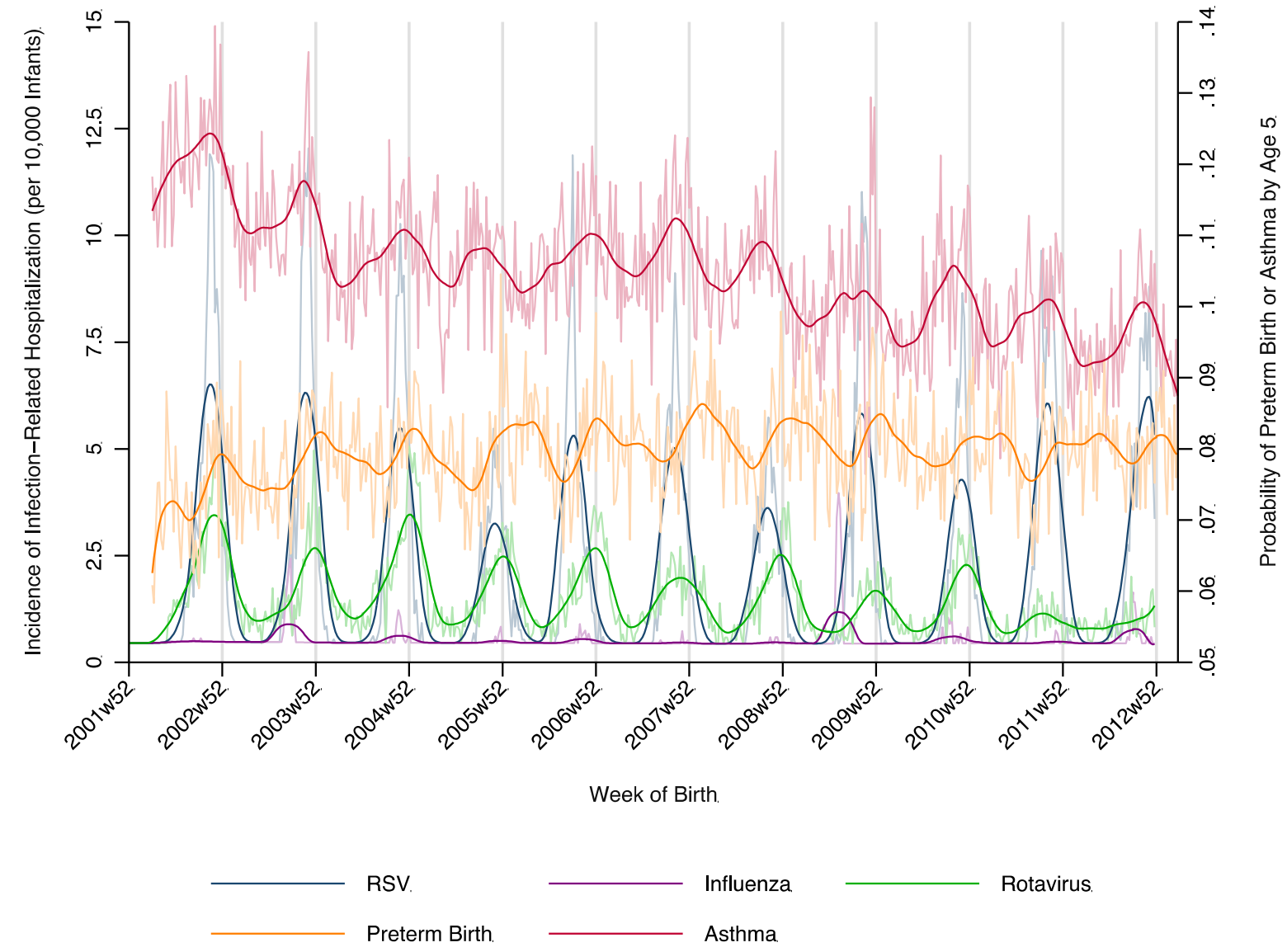

Figure S9. Incidence of RSV hospitalisation among infants by calendar week plotted alongside probability of RSV hospitalisation in the first year of life by week of birth (see Appendix 2 for definitions). Incidence of RSV hospitalisations by calendar week plotted without offset (Panel A) and with offset (Panel B) by age at which crude log-odds of asthma at age 5 was highest (i.e., 13 weeks).

A.

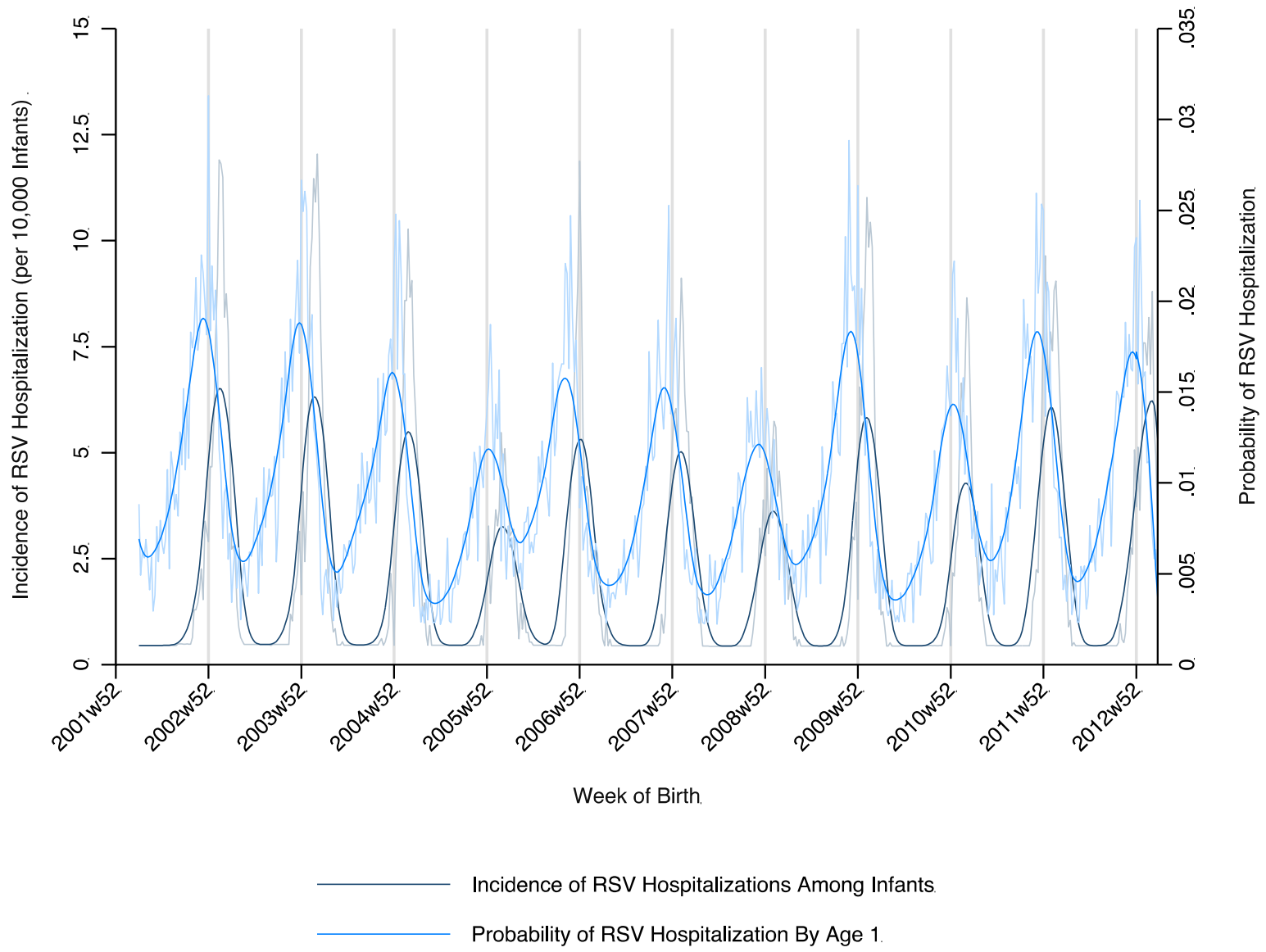

B.

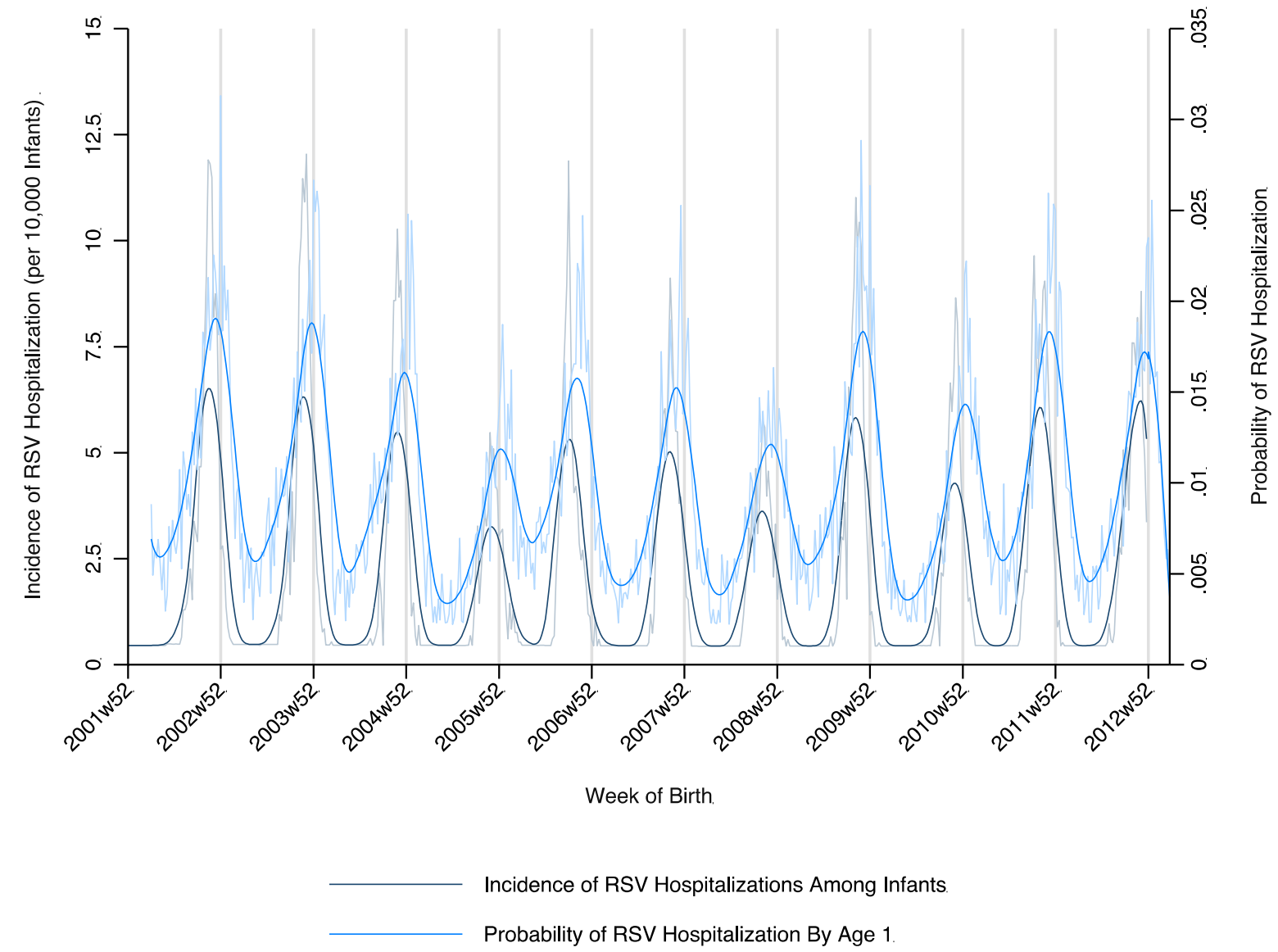

Figure S10. Incidence of influenza hospitalisation among infants by calendar week plotted alongside probability of influenza hospitalisation in the first year of life by week of birth (see Appendix 2 for definitions). Incidence of influenza hospitalisations by calendar week plotted without offset (Panel A) and with offset (Panel B) by age at which crude log-odds of asthma at age 5 was highest (i.e., 11 weeks). To facilitate visualisation in figures, incidence of influenza hospitalisation was set to a maximum of 12.5 per 10,000 infants if greater than this value and probability of influenza hospitalisation was set to a maximum of 0.008 if greater than this value (i.e. during the 2009-2010 A/H1N1 influenza pandemic).

A.

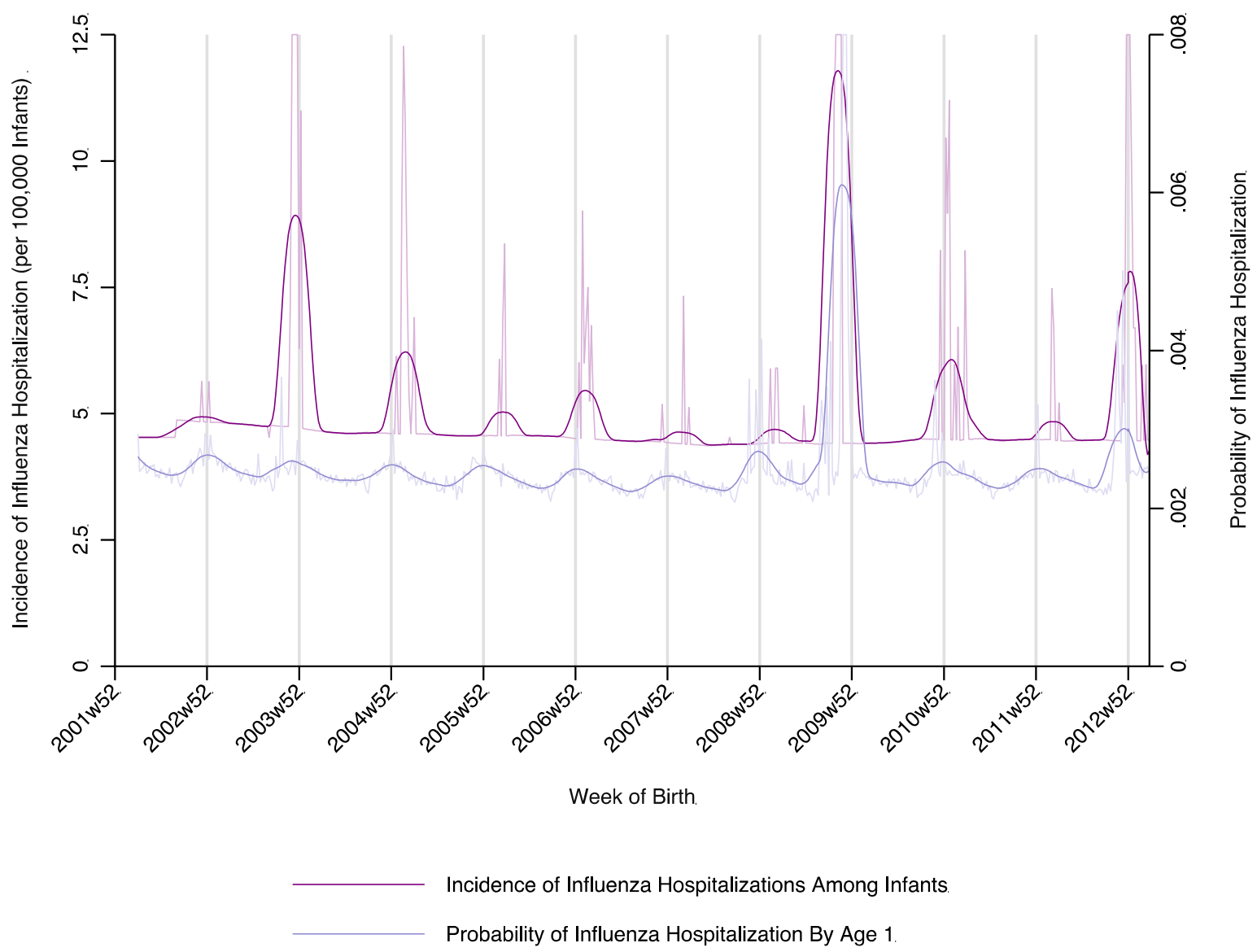

B.

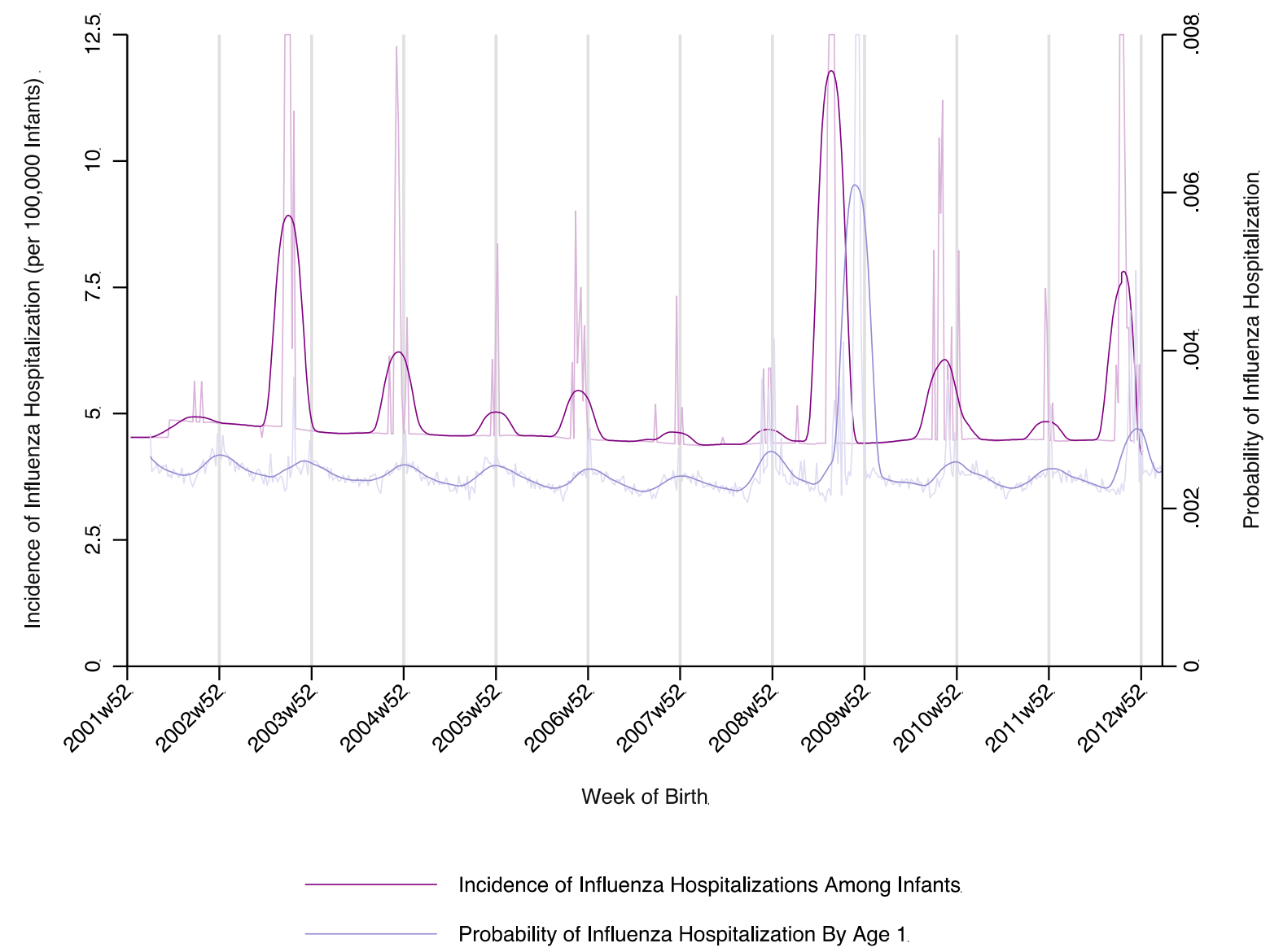

Figure S11. Incidence of rotavirus hospitalisation among infants by calendar week plotted alongside probability of rotavirus hospitalisation in the first year of life by week of birth (see Appendix 2 for definitions). Incidence of rotavirus hospitalisations by calendar week plotted without offset (Panel A) and with offset (Panel B) by age at which crude log-odds of asthma at age 5 was highest (i.e., 16 weeks).

A.

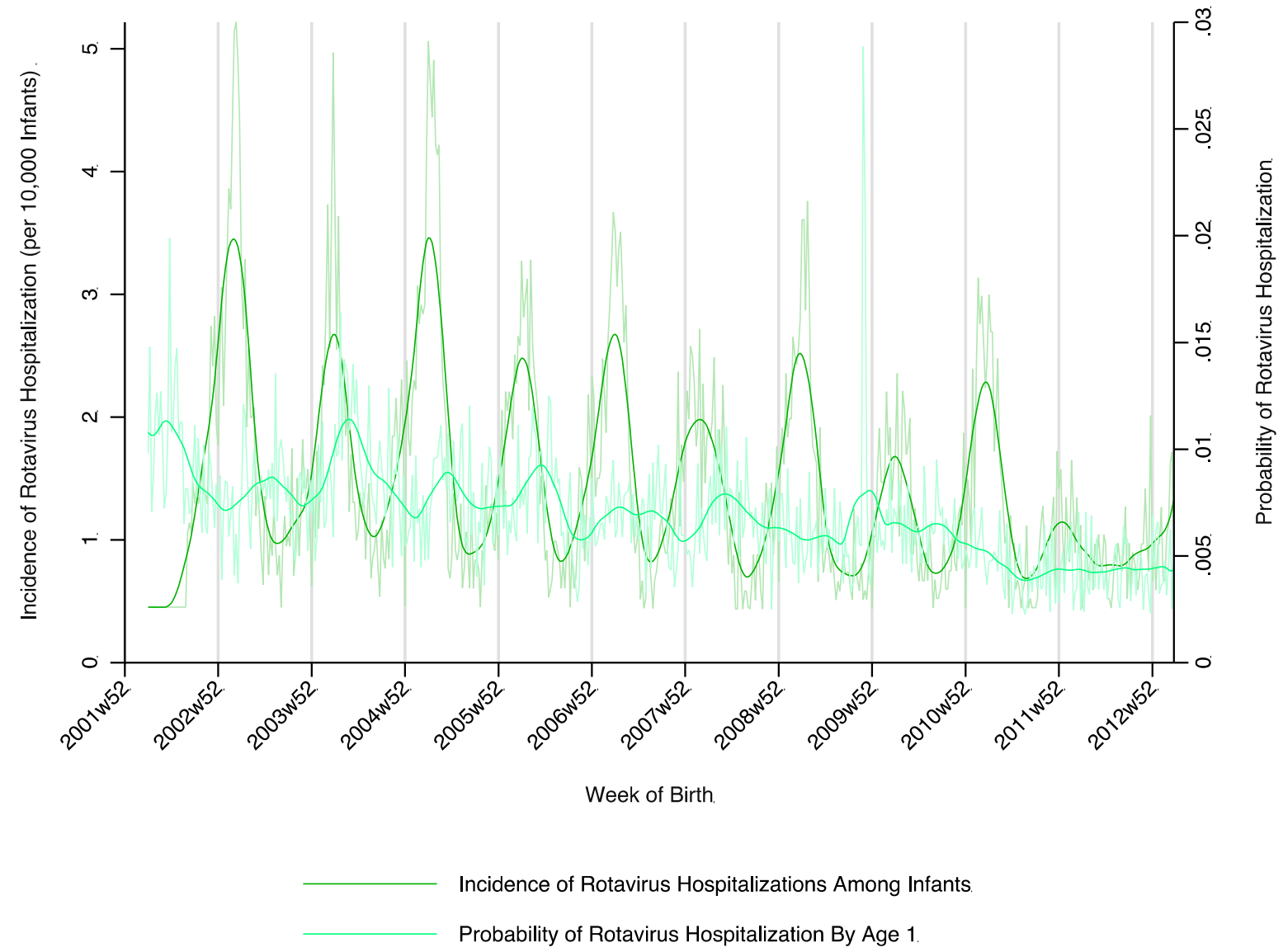

B.

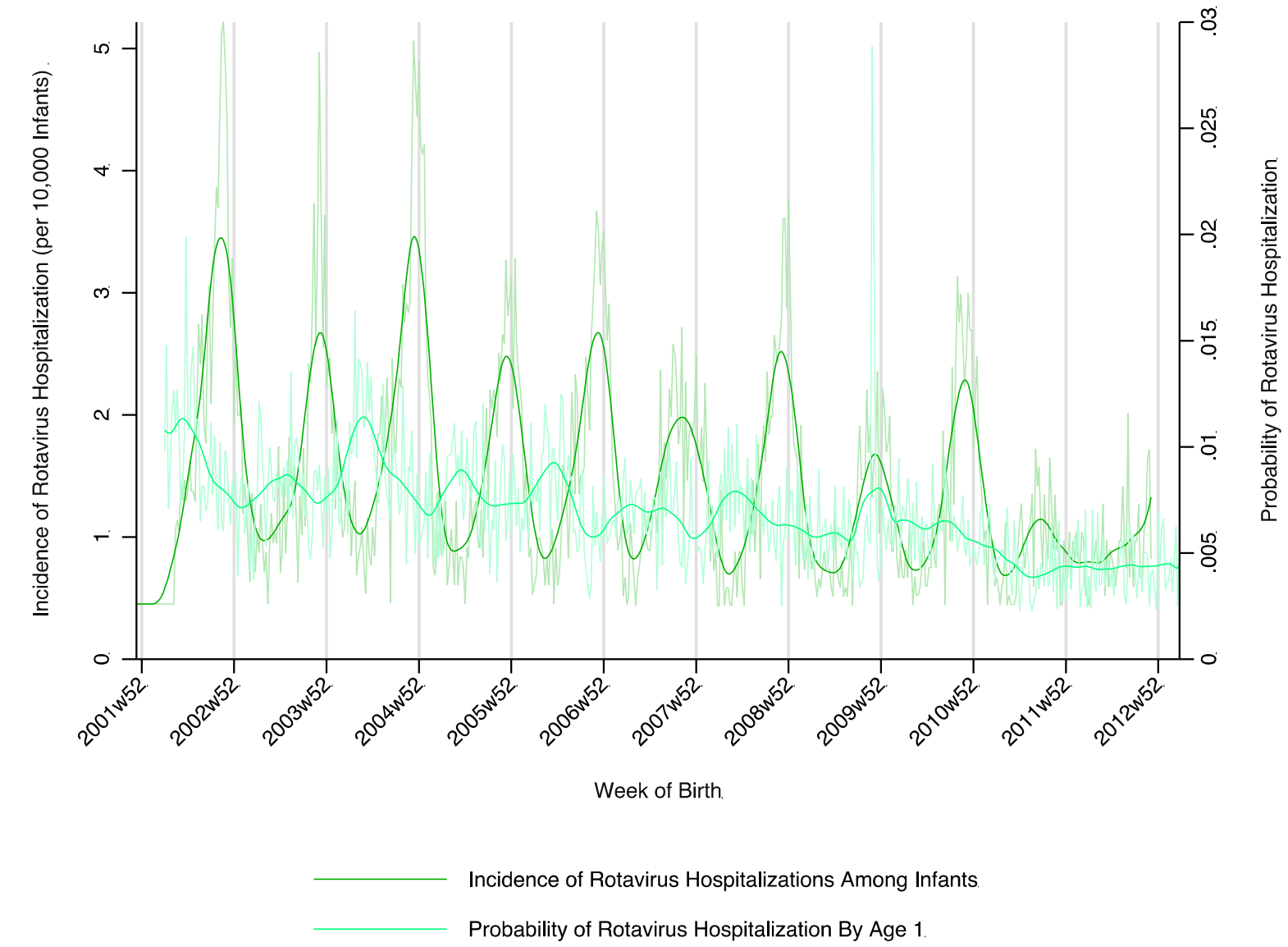

)

Table S5. Comparison of RSV measures and study population between Wu et al. and current study.

	**Wu et al.**[7]	**Current study**
Study population	Live births to Tennessee Medicaid recipients (~25% of Tennessee live births)	Live births to all Ontario residents (100% of Ontario live births)
Study size	95,310	1,437,731
RSV seasons	6 seasons[a]<br>1995-1996: 23 Dec 1995<br>1996-1997: 03 Feb 1997<br>1997-1998: 30 Dec 1997<br>1998-1999: 30 Dec 1998<br>1999-2000: 27 Jan 2000<br>2000-2001: 10 Feb 2001	11 seasons in primary analysis[b]
Infant sex; n (%)		
Female	46,615 (48.7)	700 557 (48.7)
Male	48,895 (51.3)	737 113 (51.3)
Other	0	61 (0.0)
Pregnant person smoking during pregnancy; n (%)		
No	69,602 (73.2)	
Yes	25,420 (26.8)	9.0%[c]
Gestational age at delivery (weeks)		
Median (IQR)	39 (38, 40)	39 (38; 40)
Pregnant person age (years) [d]	22	30[d]

[a]*For Wu et al., the first day of the week with the highest bronchiolitis hospitalisation frequency was considered the winter virus peak.*
[b]*For the current study, the week with the highest number of RSV hospitalisations among infants was considered the RSV peak; see Table S2 for RSV, influenza, and rotavirus seasonal peaks.*
[c]*Not available in the study dataset; estimate provided is from a provincial report of 2009 data (BORN Ontario).*
[d]*Reported as a median by Wu et al. and as a mean in the current study.*

References

1 ICES Ontario. "ICES Data Dictionary," accessed October 2019, https://datadictionary.ices.on.ca/Applications/DataDictionary/Default.aspx.

2 Canadian Institute for Health Information. "Discharge Abstract Database Metadata," accessed October 2019, https://www.cihi.ca/en/discharge-abstract-database-dad-metadata.

3 A. S. Gershon, C. Wang, J. Guan, J. Vasilevska-Ristovska, L. Cicutto, T. To, "Identifying Patients With Physician-Diagnosed Asthma in Health Administrative Databases," *Canadian Respiratory Journal* 16 (2009):183–188.

4 A. S. Gershon, J. Guan, C. Wang, T. To, "Trends in Asthma Prevalence and Incidence in Ontario, Canada, 1996-2005: A Population Study," *American Journal of Epidemiology* 172 (2010):728–736.

5 J.A. Omand, J.L. Maguire, D.L. O'Connor, et al., "Agreement Between a Health Claims Algorithm and Parent-Reported Asthma in Young Children," *Pediatric Pulmonology* 54 (2019):1547–1556.

6 T. Fitzpatrick, A.S. Wilton, A. Guttmann, "Development and Validation of a Simple Algorithm to Estimate Common Gestational Age Categories Using Standard Administrative Birth Record Data in Ontario, Canada," *Journal of Obstetrics and Gynaecology* 41 (2021):207–211.

7 P. Wu, W.D. Dupont, M.R. Griffin, et al., "Evidence of a Causal Role of Winter Virus Infection During Infancy in Early Childhood Asthma," *American Journal of Respiratory and Critical Care Medicine* 178 (2008):1123–1129.
